# Mapping the Ecology of Risk: 100 Risky Choices of Modern Life

**DOI:** 10.1177/09567976251384975

**Published:** 2025-11-06

**Authors:** Renato Frey, Olivia Fischer

**Affiliations:** Department of Psychology, University of Zurich

**Keywords:** risky choices, risk taking, risk preference, data-driven inventory

## Abstract

What are the risky choices people face in our complex and fast-changing world? This article reports on a series of population surveys in Switzerland (*N* = 4,380) that collected those risky choices that are relevant in people’s everyday lives. Using this empirical basis, we developed an inventory consisting of 100 unique real-life choices to address open questions regarding the structure, life domains, and stability of the current ecology of risk. Moreover, a follow-up study (*N* = 933) indicated some degree of generalizability of the construct of risk preference to the newly identified real-life choices. The five key insights that emerged from our analyses may be useful for researchers studying decision-making under risk and uncertainty (e.g., what criteria to use when developing novel measurement instruments) and policymaking in applied settings (e.g., addressing how swiftly the risks of modern life change).

## Introduction

In a fast-moving and globalized world, people constantly face novel challenges in their day-to-day decision-making. For instance, the latest innovations in medicine (e.g., applications of artificial intelligence; Topol, 2019), in finance (e.g., the emergence of cryptocurrencies; Pelster et al., 2019), or in communication technologies (e.g., the deployment of 5G mobile networks; Frey, 2021) implicate difficult choices between options that may have entirely unknown consequences (i.e., a decision under uncertainty)—or consequences that are known in principle but in fact are highly unpredictable because of their variability (i.e., a decision under risk). Evidently, the extent to which a person takes risks when making such a decision can substantially affect important life outcomes, for better or for worse: Whereas excessive risk taking may lead to severe harm for physical and mental well-being (e.g., Blum & Nelson-Mmari, 2004), excessive risk aversion may result in missed opportunities and stagnancy (e.g., Brunette et al., 2020).

Although it is easy and intuitive to think of examples of risky choices people have to deal with in modern life, to date no systematic examinations exist of what risky choices people perceive to be relevant and potentially challenging in their own lives, as well as in the lives of fellow citizens (for an exception focusing on big life decisions more generally, see Camilleri, 2023). This means there is a peril that theories on human decision-making are developed and tested with potentially outdated or only partially relevant real-life choices in mind. Thus, in line with repeated calls for more discovery-oriented research and a greater emphasis on description in psychological science (e.g., Cooper, 2016; Oberauer & Lewandowsky, 2019; Rozin, 2009), the main goal of this article consisted of mapping the current *ecology of risk*—that is, to analyze the landscape of risky choices that different groups of persons are confronted with in various areas of life.^1^ We did so by developing a data-driven inventory of contemporary risky choices, using large population samples and tapping people’s direct experiences and perceptions from real life. Such an inventory not only provides inherently interesting insights into the challenging choices of modern life but is also key to (a) addressing open issues in the behavioral sciences revolving around the construct of risk preference and (b) informing policymaking and applications in practice more generally.

In what follows we will report on a series of three empirical studies that all collected quota samples of the Swiss general public (Fig. 1). Studies 1 (*N* = 2,178) and 2 (*N* = 2,202) jointly collected a total of 4,380 risky choices that participants either faced in their own lives or that they believed to be relevant for a large number of fellow citizens (the latter being inspired by the idea of *social sensing*; Galesic et al., 2021). We used different subsets of these choices to develop an inventory of risky choices following a multistage process, which included a separate discovery phase (Stage A1) and a preregistered validation phase (Stage A2). We then scrutinized the resulting inventory of risky choices using a set of preregistered analyses (Stage B), aimed at addressing the first three of our main research questions (RQs) introduced below. Study 3 (*N* = 933) consisted of an independent follow-up assessment of the newly developed inventory of risky choices and was aimed at addressing our fourth RQ by means of a further set of preregistered analyses (Stage C).

**Fig. 1.**
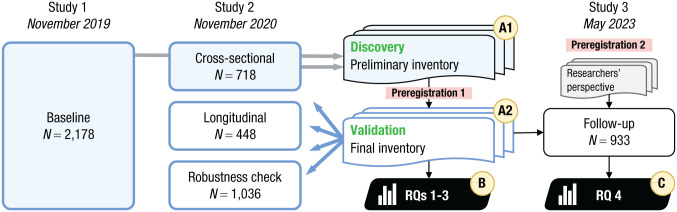
Overview of the different studies and the multi-stage process of the inventory development, including the discovery phase (stage A1) and separate validation phase (stage A2). In each phase, two independent raters processed the risky choices as reported by participants using a dedicated web app (Fig. S1). The newly developed inventory was used to address our main RQs 1–3 (stage B; preregistered), and finally we conducted a follow-up study to address RQ 4 (stage C, preregistered).

In RQ 1, we examined what choices the current ecology of risk involves. We addressed this RQ by mapping the most commonly reported risky choices for all participants of our samples (RQ 1a) as well as for different subgroups of the population (RQ 1b). In RQ 2 we used the newly developed inventory to scrutinize the appropriateness of various life domains that are commonly used in behavioral decision research. We addressed this RQ by analyzing the distributions of risky choices to different life domains for all participants of our samples (RQ 2a) and for different subgroups of the population (RQ 2b). Furthermore, to clarify whether these life domains can adequately distinguish between different real-life risky choices, we examined the domain homogeneity of risky choices and potential co-occurrence of life domains (RQ 2c). In RQ 3 we adopted a dynamic perspective and assessed how stable versus transient the risky choices are that people are confronted with at different points in time, such as during a global state of emergency. We addressed this RQ by comparing the ecology of risk before and during the COVID-19 pandemic, both in terms of the actual risky choices (RQ 3a) and their associated life domains (RQ 3b). Finally, in RQ 4 we examined how frequently each of the choices of the newly developed inventory is encountered in everyday life (RQ 4a). In doing so, as a benchmark we used a set of prototypical risky choices often investigated in behavioral decision research, thus representing the researchers’ perspective. Moreover, we examined the generalizability of the construct of risk preference by testing the extent to which the latter is associated with how often a person chose the risky options in the various choices (RQs 4b and 4c).

## Research Transparency Statement

### General disclosures

**Conflicts of interest:** Both authors declare no conflicts of interest**. Funding:** This research was supported by the Swiss National Science Foundation (Grant Nos. PZ00P1_174042 and PCEFP1_194540). **Artificial intelligence:** No artificial-intelligence-assisted technologies were used in this research or the creation of this article. **Ethics:** This research received approval from the ethics committee of the Faculty of Psychology, University of Basel (Studies 1 and 2: No. 021–19–2) and from the ethics committee of Faculty of Arts and Social Sciences, University of Zurich (Study 3: No. 23–04–12). **Open Science Framework (OSF):** To facilitate long-term preservation, we registered all OSF files at https://osf.io/gcfz5. **Computational reproducibility:** The computational reproducibility of the results in the main article (but not the supplementary materials) has been independently confirmed by the journal’s STAR team.

### Studies 1 and 2 disclosures

**Preregistration:** After the data collection and once the discovery phase (A1) of the inventory-development process was completed (see Fig. 1), the preliminary inventory, the research questions, the methods for the inventory validation (A2), and the analysis plan were preregistered at https://osf.io/kbs5u/ (October 12, 2021). There were minor deviations from the analysis plan in how we addressed RQ 3 (see Section III.a in the Supplemental Material available online). **Materials:** All study materials are publicly available. The instructions are reported in the Method section, and the materials processed during the inventory development are available in the data set. The code base of the study is available at https://osf.io/m9t5v/. **Data:** All primary data are publicly available at https://osf.io/m9t5v/. **Analysis scripts:** All analysis scripts are publicly available at https://osf.io/m9t5v/.

### Study 3 disclosures

**Preregistration:** Prior to data collection, the research questions, methods, and the analysis plan were preregistered at https://osf.io/kthpr/ (May 2, 2023). There were minor deviations from the analysis plan in how we addressed RQ 4 (see Section III.b in the Supplemental Material). **Materials:** All study materials are publicly available. The instructions are reported in the Method section, and the items are contained in the data set. The code base of the study is available at https://osf.io/m9t5v/. **Data:** All primary data are publicly available at https://osf.io/m9t5v/. **Analysis scripts:** All analysis scripts are publicly available at https://osf.io/m9t5v/.

## Method

### Overview of samples and participant characteristics

We collected a total of five samples across three studies (see Fig. 1). Table S1 in the Supplemental Material provides an overview of the five samples and the respective sociodemographic characteristics of participants (all participants were German or Swiss-German speakers). In Study 1, conducted in November 2019 (i.e., before the outbreak of the COVID-19 pandemic), we collected the baseline sample—that is, a large sample of the Swiss general public (*N* = 2,178), with quota matching for gender and four age groups (15–29 years; 30–44 years; 45–59 years; 60–79 years). No upper limit concerning age was enforced, which is why the age of some participants exceeded the indicated age range of these quotas. Study 2 was conducted in November 2020 (i.e., approximately 1 year later and during the COVID-19 pandemic) and collected three samples in parallel: Participants of the longitudinal sample (*N* = 448) were part of the baseline sample and were invited to participate again. Conversely, participants of the cross-sectional sample (*N* = 718) and the robustness-check sample (*N* = 1,036) were newly invited, using the same quotas as specified for Study 1. (See the Materials and Procedures section of Studies 1 and 2 for the difference between the cross-sectional sample and the robustness-check sample.)

Study 3 was conducted in May 2023 and collected the follow-up sample, that is, an independent sample of the Swiss general public (*N* = 933), with quota matching for gender and four age groups (18–29 years; 30–44 years; 45–59 years; 60–79 years). No upper limit concerning age was enforced, which is why the age of some participants exceeded the indicated age range of these quotas.

Participant recruitment was performed by an external survey company using an existing panel, which exclusively comprised participants who were actively recruited by telephone (i.e., no self-registrations possible). The sample sizes of the various studies were based on recommendations by the survey company for robustly covering all of the specified quotas. The ethics committee of the Faculty of Psychology, University of Basel, approved Studies 1 and 2 (No. 021–19–2), and the ethics committee of the Faculty of Arts and Social Sciences, University of Zurich, approved Study 3 (No. 23–04–12).

### Materials and procedures in Studies 1 and 2

The studies were run online using a mobile-first design (i.e., the studies could be completed on all types of devices), and the procedures concerning the collection of informed consent, sociodemographic data, and risky choices were identical in both studies (except for the robustness-check sample; see next section). Participants were compensated according to the incentive scheme of the survey company, namely, with 300 points that they could use at their own discretion (e.g., convert into a monetary compensation of 3 CHF (Swiss francs); convert into a donation; or convert into various shopping vouchers).

#### Onboarding

Participants were first informed about the background and purpose of the study and then provided informed consent. Next, they reported sociodemographic information (i.e., gender, age, canton of residence, postal code, level of education, employment status). Participants in Study 1 and participants in the longitudinal sample and the cross-sectional sample of Study 2 were part of another study on individual differences in risk perceptions; they next completed a series of questions unrelated to the current research questions (i.e., regarding their perceptions of 5G, the latest generation of cellular network technology; Frey, 2021). This block was omitted for participants of the robustness-check sample, who directly proceeded to the main task (see Section II.a in the Supplemental Material).

#### Main task: reporting of risky choices

In the main task of Studies 1 and 2, each participant reported one real-life risky choice. In order to obtain a diverse set of risky choices, including relatively frequent choices most people are confronted with, as well as relatively rare choices not everyone may be confronted with on a regular basis, we randomly assigned participants to one of four conditions using a two-by-two between-subjects design: The first factor varied whether participants were prompted to think of a risky choice from their personal life (*personal* perspective) or a risky choice many other people have to make (*other* perspective). The latter was inspired by the idea of *social sensing*, that is, the observation that “people seem to have a good grasp of the relative frequency distributions of different properties of their immediate social circles” (Galesic et al., 2021, p. 216). Specifically, rather than only prompting people to recall risky choices that they experienced personally, it may be helpful tap the risky choices they have seen other people in their social environment make. This approach may counteract the reporting of highly idiosyncratic choices as well as potentially reduce some factors that can bias responses, such as social desirability. The second factor varied whether participants were instructed to think of a risky choice in which they (i.e., for the *personal* perspective) took a risk (*risk taken*) versus did not take a risk (*risk not taken*). Accordingly, for the other perspective participants were instructed to think of a risky choice in which many other people take a risk (*risk taken*) versus do not take a risk (*risk not taken*). Specifically, the instructions were as follows (translated from German into English; see Section I in the Supplemental Material for the original wording):

People greatly differ in how they deal with the opportunities and risks of modern life.

[*Personal* perspective]: Now please try to **remember an important decision from your own life** where you had the choice to either take a risk or avoid it—and you ultimately decided **in favor of [against] the risk**.

[*Other* perspective]: Now please think of an **important decision that many people have to make** and where people have the choice to either take a risk or avoid it. Please think of a decision where, in your opinion, most people ultimately decide **in favor of [against] the risk**.

To facilitate the response process and to make sure participants entered a risky choice that consisted of two choice options, we displayed a text format with blank spaces that participants had to fill in. Specifically, participants saw the following text (illustration for the condition *personal* perspective/*risk taken*), with two empty placeholders for the risky and the safe options, including a summary sentence in the end that was automatically updated with the respective option, depending on what participants typed in:

I had to decide whether to either take a risk and [risky option] or avoid a risk and thus [safe option].

In this decision, I ultimately decided **in favor of the risk**, namely that I would [risky option].

Taken together, we aimed to collect risky choices in a way as structured as possible—for example, by ensuring that the choices consisted of a relatively risky and a relatively safe option, and by using a two-by-two between-subjects factor design to sample various risky choices and reduce response bias as much as possible. However, we did this without constraining participants from thinking freely about what they have experienced as risky choices or what they consider to be risky choices for other people in real life.

On the next page, participants were shown the risky choice they had just typed in and were asked to choose from a drop-down menu the life domain that best captured this choice. The options were taken from the German Socio-Economic Panel (SOEP; e.g., Falk et al., 2016) and consisted of financial, health, recreational, occupational, social, and traffic domains (translated from German into English). Furthermore, participants were given the option to type in an additional life domain (free text input) if they felt that none of these options optimally captured the life domain of their risky choice.

### Analysis plan and preregistration of Studies 1 and 2

Our analysis plan involved two major stages and is reported in full detail in our preregistration at https://osf.io/kbs5u/. In Stage A, we developed the inventory of risky choices. This stage consisted of a discovery phase (Stage A1) and a separate validation phase (Stage A2). During the discovery phase, multiple research assistants (i.e., raters) sequentially screened a subset of the reported risky choices and registered a new inventory choice whenever they could not assign a choice to one of the already-existing inventory choices. Then the preliminary lists of inventory choices were merged and consolidated (see preregistration for detailed procedures). During the subsequent validation phase, new research assistants (i.e., independent raters) classified all of the reported risky choices to the preliminary inventory choices (see preregistration for detailed procedures). Finally, in Stage B we ran a series of preregistered analyses on the resulting inventory to address RQs 1 through 3. All analyses were conducted in *R* version 4.2.1 (R Core Team, 2019). The data and full analysis code are provided at https://osf.io/m9t5v/.

In order to restrict researchers’ degrees of freedom and increase the robustness of the inventory of risky choices and the results regarding our main research questions, we submitted our preregistration after having completed Stage A1. Specifically, we registered the intermediate results of the discovery phase of the inventory development, and we preregistered the analysis plan for Stage A2 (i.e., the validation phase of the inventory development) and Stage B (i.e., the analyses of our main research questions).

Moreover, to streamline the inventory-development process, we developed and used a dedicated web application (see Fig. S1 in the Supplemental Material). In Stage A1 of this process (discovery), the app enabled the raters to either select one of the already existing inventory choices to classify a participant selection, or to register a new inventory choice—provided that a rater concluded that none of the existing inventory choices were suitable to this end. In Stage A2 (validation), the independent raters could select only from the inventory choices of the preliminary inventory to classify the participant choices.

### Inventory development: from participant choices to inventory choices

The classification of participant choices to inventory choices unfolded as follows: During the discovery phase (Stage A1) and starting from a subset of 2,896 risky choices (participants of the baseline sample and the cross-sectional sample), two raters assembled a preliminary inventory consisting of 157 presumably unique risky choices. These *inventory choices* formed a hierarchical structure consisting of *generic* choices (e.g., quit one’s job vs. not quit one’s job) and *subordinate* choices that contained additional explanations and qualifiers (e.g., quit one’s job vs. not quit one’s job and be unhappy). With these 157 inventory choices, 1,760 (89%) of the 1,968 participant choices that were identified as valid (77%) could be classified (see the preregistration for the exact criteria and detailed procedures; the detailed classification of the original participant choices to the inventory choices can be inspected in the data set).

Subsequently, during the preregistered validation phase (Stage A2), two independent raters classified the full 4,380 participant choices stemming from all four samples of Studies 1 and 2 (also including the choices of the hold-out samples, which were not included during the discovery phase). Using 156 of the 157 preliminary inventory choices, the two raters classified 3,374 (77%) of all participant choices unanimously as the same inventory choice. For the remaining 1,006 (23%) of the participant choices that were classified differently by the two raters, a third, independent rater adopted the role of a tiebreaker. The raters assigned the participant choices to the 156 inventory choices with a very high overall classification confidence, namely, a mean confidence of 91.5 on the scale ranging from 0 to 100 (Fig. S2 in the Supplemental Material). When collapsing the subordinate choices into their respective generic choices, 112 generic inventory choices remained.

Finally, to derive the final set of risky choices for the inventory we applied our preregistered criteria: First, we retained only inventory choices to which at least three participant choices were assigned. This reduced the set of inventory choices to 102 choices, which still accounted for 2,740 of the 2,758 participant choices that were identified as valid. Second, all of the inventory choices had a classification confidence higher than 50, and thus no inventory choice had to be removed according to our second preregistered criterion. Third, we removed the two inventory choices with the lowest classification confidence to arrive at 100 inventory choices, which still accounted for 2,729 of all valid participant choices.

### Materials and procedures in Study 3

Like Studies 1 and 2, the follow-up Study 3 was run online using a mobile-first design (i.e., the study could be completed on all types of devices). A total of 1,030 participants completed the study. After removing one participant who used a web service to translate the online study into Polish, and after removing 37 participants who did not fulfill our preregistered criterion of answering at least two of the three attention checks correctly, the sample size consisted of 992 participants. We also removed the data of 59 participants who reported excessively high frequencies of having encountered the various risky choices (see Section III.b in the Supplemental Material), resulting in the final sample size of 933 participants.

Using a between-subjects design, we randomly assigned participants to one of two orders of the main tasks—reporting risk preferences and then reporting the encountering of risky choices, or vice versa. Participants were compensated according to the incentive scheme of the survey company, namely, with 100 points that they could use at their own discretion (e.g., convert into a monetary compensation of 1 Swiss franc, or CHF; convert into a donation; or convert into various shopping vouchers).

#### Onboarding

Participants were first informed about the background and purpose of the study and then provided informed consent. Next, they reported sociodemographic information (i.e., gender, age, level of education, employment status, yearly gross income) and the device used to participate in the study (i.e., computer/laptop, tablet, smartphone).

#### Reporting the encountering of risky choices in real life

We informed participants that they were about to see a number of risky choices that they might have encountered once or more in the past and that we would ask two questions for each of these choices (translated from German into English):

1. Frequency: First, we will always ask how often you have had to make this decision in the past—regardless of whether you decided for or against the activity shown. By default we will ask for the frequency “in the last month,” but you are free to choose the time period that allows you to provide the most precise information possible.
2. Chosen option: We will then ask you whether you have opted for or against the activity mentioned in the past. If you have never had to make the decision before, we will ask you how you would make this decision (i.e., hypothetically) if you were in this situation yourself in the future.

Participants were then presented with a number of risky choices in a randomized order. On the one hand, these comprised the 100 risky choices of the inventory newly developed in Studies 1 and 2—thus representing the layperson’s perspective on the ecology of risk. (Note that we originally also presented two additional choices that were only part of the preliminary inventory and which were thus not further analyzed; see Section III.b in the Supplemental Material). On the other hand, these choices also comprised 62 risky choices commonly used as frequency measures in behavioral research on people’s risk preferences (i.e., “How often did you do . . . in the past?”)—thus representing the researchers’ perspective on the ecology of risk. We assembled the latter choices in a separate project using a three-step process (for all methodological details, see https://osf.io/qyjw8/): First, by means of a literature review, we identified a total of 620 risky choices in the form of frequency measures. Second, this initial set was supplemented by 24 additional risky choices that were identified in an online study, which prompted researchers of the behavioral sciences (via requests to mailing lists) to contribute additional measures potentially not yet part of the initial set. Third, we systematically consolidated these risky choices (e.g., collapsing identical choices, only retaining choices that appeared at least three times) into a final set of 63 unique choices. In Study 3 we excluded one choice (“to attempt suicide”) because of its potentially triggering effect on participants, leaving 62 choices.

For each of the total of 162 risky choices we displayed only the risky option because the safe option typically consisted of the inverse of the former (i.e., not doing the risky option). Note that this does not imply that the risky option always coincided with performing an action, as some risky options also consisted of inaction (e.g., “not get insurance”). Participants could first choose the reference class that permitted them to indicate the most precise frequency estimate for the shown option (i.e., in the last week, month, year, 10 years, 50 years), and then typed in an integer indicating how often they were confronted with the choice of whether to engage in the described activity within the selected interval. Provided that they typed in a number larger than zero, they were asked how they usually decided in the past, namely, *more for it* (i.e., risk taken) versus *rather against it* (i.e., risk not taken). Alternatively, and provided that participants typed in zero (i.e., never encountered the current choice in the past), they were asked (with the same response options as those just introduced), “If you ever had to make this decision in the future, would you rather choose to do this activity or not?”

We interspersed three attention checks that prompted participants to type in a specific frequency and select a specific option. Moreover, once a participant had completed 80 choices (i.e., approximately half of the choices) they were prompted to take a break and could only continue after at least 5 min.

#### Reporting of risk preferences

The assessment of participants’ risk preferences always started with an item measuring risk preferences in general (i.e., “How willing are you to take risks in general?”), followed by six items measuring domain-specific risk preferences (i.e., “How would you rate your willingness to take risks with regards to traffic / financial investments / recreation and sports / your professional career / health / trust in strangers?”). The latter items were presented in a randomized order. Participants responded to all items using a visual analogue scale, that is, a continuous slider with the endpoints labeled *not willing to take risks at all* and *very willing to take risks*, respectively, and the midpoint labeled *neither* (no position on the slider was preselected). The slider recorded a response value ranging from 0 to 100. All items were taken and adapted from the preference survey module of the SOEP (Falk et al., 2016).

#### Analysis plan and preregistration of Study 3

The analyses of Study 3 constituted our final analysis stage (Stage C). We preregistered the respective analysis plan at https://osf.io/kthpr/ (note that RQs 1–3 of the preregistration correspond to RQs 4a–c here). All analyses were conducted in *R* version 4.2.1 (R Core Team, 2019). The data and full analysis code are provided at https://osf.io/m9t5v/.

## Results

### RQ 1: What are the most prominent risky choices people are confronted with in modern life?

Figure 2 depicts a word cloud of all 4,380 risky choices as reported by participants of Studies 1 and 2 (i.e., participant choices). On the basis of these choices, we developed the inventory of 100 risky choices. To address our first RQ, we examined the frequencies with which the various risky choices of the inventory were reported by participants. We first did so at the population level (RQ 1a)—that is, by analyzing the data of all unique participants of Studies 1 and 2, excluding participants of the longitudinal sample. Specifically, Figure 3 depicts the 25 most frequently reported risky choices, and Figures S3 to S5 depict the 75 remaining risky choices of the full inventory. As can be seen, the most frequently reported choices of the current ecology of risk were whether to accept a new job (229 participants reported this choice), whether to quit one’s job (i.e., without the prospect of a new job; 211 participants reported this choice), whether to invest money (148 participants reported this choice), whether to drive a car (133 participants reported this choice), whether to become self-employed (130 participants reported this choice), and whether to buy a house (96 participants reported this choice). Figure 4 illustrates the words associated with these top six risky choices as expressed in participants’ original reports (translated from German).

**Fig. 2.**
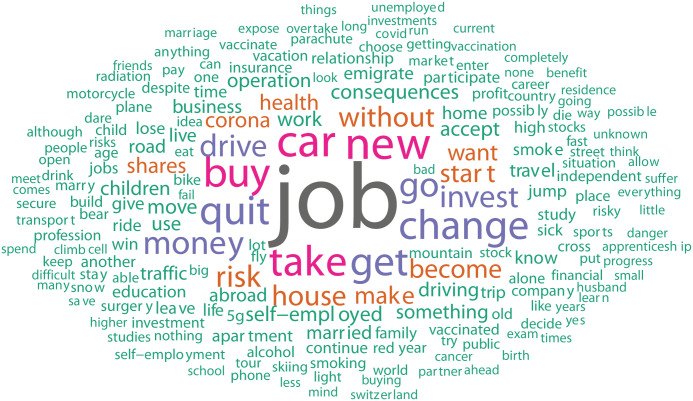
Word cloud of all individual risky choices as reported by participants in Studies 1 and 2 (*N* = 4,380).

**Fig. 3.**
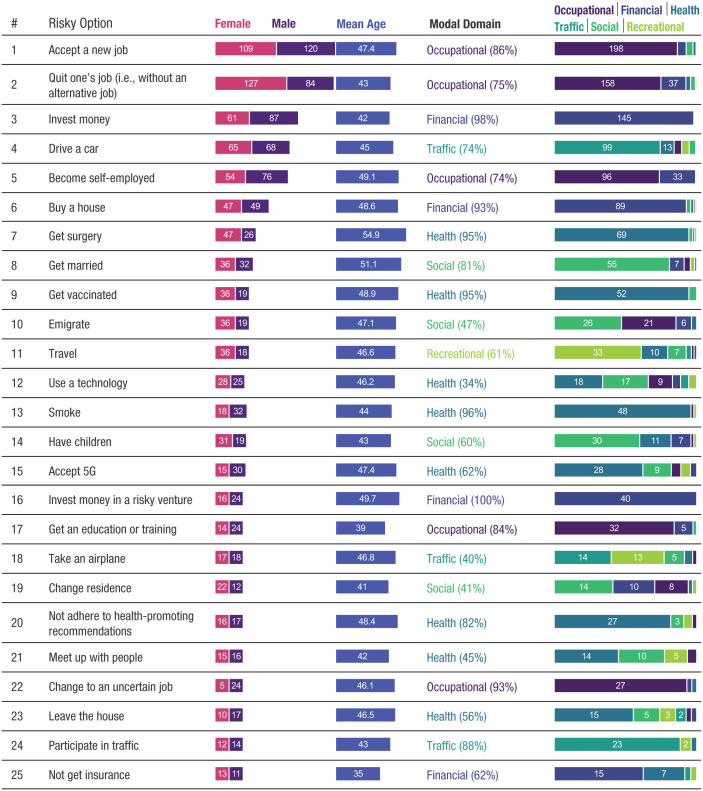
The ecology of risk: the 25 most frequently reported risky choices, along with how many women and men reported a choice, the mean age of participants reporting a choice, the domain a choice was assigned to by participants most of the time (including the percentage of all assignments), and the exact number of assignments to each domain (indicating a choice’s domain homogeneity). See Figures S3, S4, and S5 in the Supplemental Material for the 75 remaining risky choices of the full inventory.

**Fig. 4.**
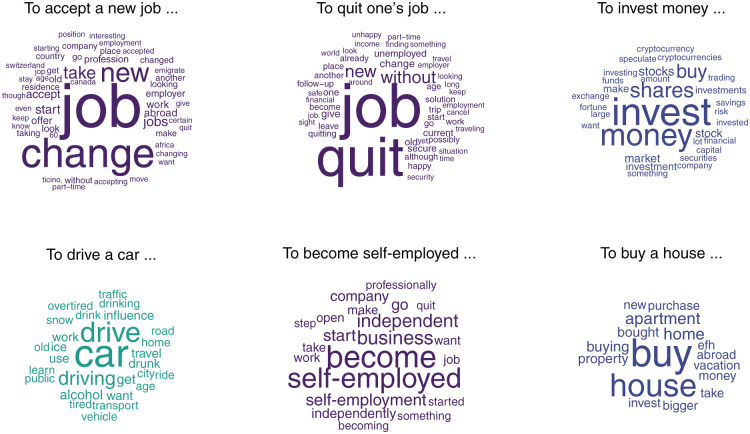
Words associated with the six most frequently reported risky choices.

When breaking down the overall inventory by eight subgroups of the population (RQ 1b), that is, by crossing gender (female, male) and four age groups (15–29 years, 30–44 years, 45–59 years, and 60 years and older), the overall pattern of the inventory by and large persisted (Tables S2 and S3). Yet some instructive changes did emerge: For instance, for both women and men, with increasing age the choice of whether to quit one’s job became less prevalent as opposed to accepting a new job. Moreover, some choices that were very prominent for one gender were not among the top five for the other gender. For example, although women in two age groups frequently considered whether to get a certain education or training (age groups 15–29 years and 45–59 years) or whether to get married (age groups 30–44 years and 60+), these two choices did not appear in the five most frequent choices for men. Conversely, although men frequently mentioned considering travel (30–44 years), getting surgery (30–44 years), and accepting 5G (60+), these choices did not appear in the five most frequent choices for women.

### RQ 2: In which life domains do the most prominent risky choices occur?

To address our second RQ, we examined the distribution of risky choices to different life domains. We first did so again at the population level (RQ 2a), and the results suggest that the newly developed inventory spans relatively broadly the six life domains typically investigated in research on risky choice: Specifically, 771 (32%) risky choices were reported to have occurred in the occupational domain, 443 (18%) in the health domain, 403 (17%) in the financial domain, 317 (13%) in the social domain, 295 (12%) in the traffic domain, and 209 (9%) in the recreational domain (see Fig. S6 and Section II.b in the Supplemental Material).

We also examined the prevalence of different life domains for different subgroups of the population (RQ 2b). The distributions of inventory choices to different domains varied somewhat across gender and age groups (Fig. S7). For instance, for persons in the age groups of 15 to 29 years, 30 to 44 years, and 45 to 59 years, the occupational domain tended to capture the most risky choices, whereas persons in the age group 60+ years, particularly women, reported risky choices about equally often in the occupational and health domains. Also, except for the age group 30 to 44 years, women tended to report more choices in the social domain than men.

Next, to evaluate whether these frequently researched life domains can adequately distinguish between different real-life risky choices (RQ 2c), we first computed domain homogeneity scores as a diversity index for each inventory choice (i.e., the relative number of participant choices with the same domain as the modal domain of the assigned inventory choice; this score was highly correlated with Shannon entropy, *r* = −.94). Figure S8 in the Supplemental Material shows the distributions of these scores, separately for inventory choices with the same modal life domain. Each horizontal line represents an inventory choice’s domain homogeneity, with a score of 100% indicating that all participant choices assigned to this inventory choice had the same life domain, whereas a score of 0% would, in principle, indicate the opposite. As can be seen, the inventory choices had a relatively large degree of domain homogeneity: Overall, 21 of the 100 inventory choices had a score of 100% (i.e., perfect domain homogeneity). Separately, for the inventory choices of the six domains, the mean homogeneity scores were 80% (occupational), 72% (health), 84% (financial), 73% (social), 82% (traffic), and 76% (recreational). Overall, 78% of the participant choices assigned to a specific inventory choice had the same life domain.

### RQ 3: How stable is the ecology of risk, such as during a global state of emergency?

To address our third RQ, we examined the stability of the ecology of risk in the wake of a global shock, namely the COVID-19 pandemic. That is, we aimed to investigate whether such an incisive occurrence drastically changed the types of risky choices people were confronted with. We did this on the level of individual risky choices (RQ 3a) and life domains (RQ 3b), and comparing the reported choices and domains in the baseline sample to both the cross-sectional and longitudinal samples.

On the level of individual risky choices (RQ 3a), we estimated Bayesian Spearman rank correlations (see Section II.a in the Supplemental Material) regarding how often each of the 100 inventory choices occurred in the different samples (i.e., counted as the total number of choices reported by participants that were assigned to the 100 inventory choices). This analysis revealed a considerable stability in the frequencies of inventory choices from the time before COVID-19 in the baseline sample to during COVID-19 in both the cross-sectional and longitudinal samples, with Spearman rank correlations of .64 (95% highest density interval [HDI] = .51, .74) and .69 (95% HDI = .57, .78), respectively. Similarly, when focusing only on the 10 most frequently reported risky choices in the different samples, there was also a high stability in the respective proportions from before to during COVID-19 (see Section II.c and Fig. S9 in the Supplemental Material for details).

Furthermore, we also examined the consistency of the risky choices’ life domains from before to during the pandemic (RQ 3b). Specifically, despite the relative stability on the level of individual risky choices, the pandemic might have brought about a systematic shift in the life domains in which these choices unfolded. Yet we found that the proportions of the risky choices’ domains did not credibly differ from before the COVID-19 pandemic to during the pandemic, either when comparing the baseline to the cross-sectional sample or when comparing it to the longitudinal sample. Figure 5 illustrates these results and shows the distribution of reported risky choices to the different domains, with the overlapping 95% HDIs and the Bayes factors (BFs) indicating that there were no credible shifts from before to during the pandemic. There were some trends indicating that the proportion of the reported risky choices in the health domain increased and the proportion of the reported risky choices in the occupational domain decreased during COVID-19 (both in the cross-sectional and longitudinal samples), but according to the 95% HDIs and the BFs, there were no credible changes from the baseline assessment.

**Fig. 5.**
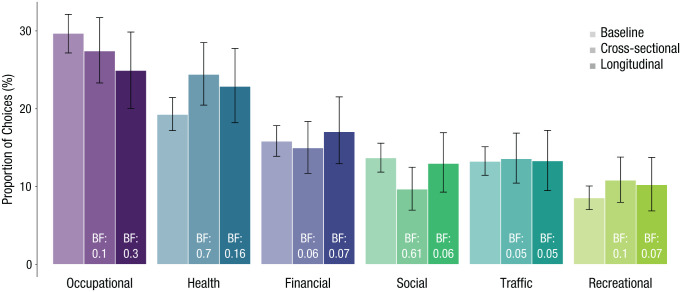
Distributions of the risky choices’ life domains as a function of the different samples. The error bars represent 95% HDIs of the estimated proportions; Bayes factors (BFs) indicate the evidence that a proportion differs from the equivalent proportion in the baseline sample (values smaller than 1 indicate that there is no difference).

### RQ 4: How prevalent are the risky choices of the new inventory, and do people’s risk preferences generalize to their actual choice behaviors?

To address our fourth and final set of RQs, we first examined how frequently participants of an independent follow-up study (i.e., Study 3) had previously experienced each of the 100 choices of the new inventory (RQ 4a). As outlined in the introduction, we compared the prevalence of these risky choices (i.e., reflecting the layperson’s perspective on the ecology of risk) with the prevalence of 62 risky choices often used as frequency measures in behavioral decision research (i.e., reflecting the researchers’ perspective on the ecology of risk).

The results indicate that participants on average had encountered the former risky choices 45 times per year and the latter risky choices only 26 times per year. Comparing only the 25 most common risky choices of the two inventories, participants on average encountered the choices of the newly developed inventory 168 times per year (Fig. 6a) and the choices typically studied in behavioral decision research only 60 times per year (Fig. 6b). To quantify the differences between the two perspectives, we implemented a Bayesian mixed-effects model with the frequency of encountering a risky choice as the dependent variable; the inventory of risky choices (laypersons’ perspective vs. researchers’ perspective) as an independent variable; and gender, age, and position of presentation—that is, the position at which a choice was presented to a participant during the study—as covariates (see Section II.d in the Supplemental Material for full model specifications). This model suggested a mean difference in the frequency of encountering a risky choice (i.e., between the two perspectives) of 33.4 (95% HDI = 27.9, 40). Moreover, an increase of 1 *SD* in age was associated with a decrease of −11.1 (95% HDI = −12.2, −9.7) in the frequency of encountering a risky choice. The frequency of encountering a risky choice was not associated with gender or position of presentation. All model parameters are reported in Table S4 in the Supplemental Material.

**Fig. 6.**
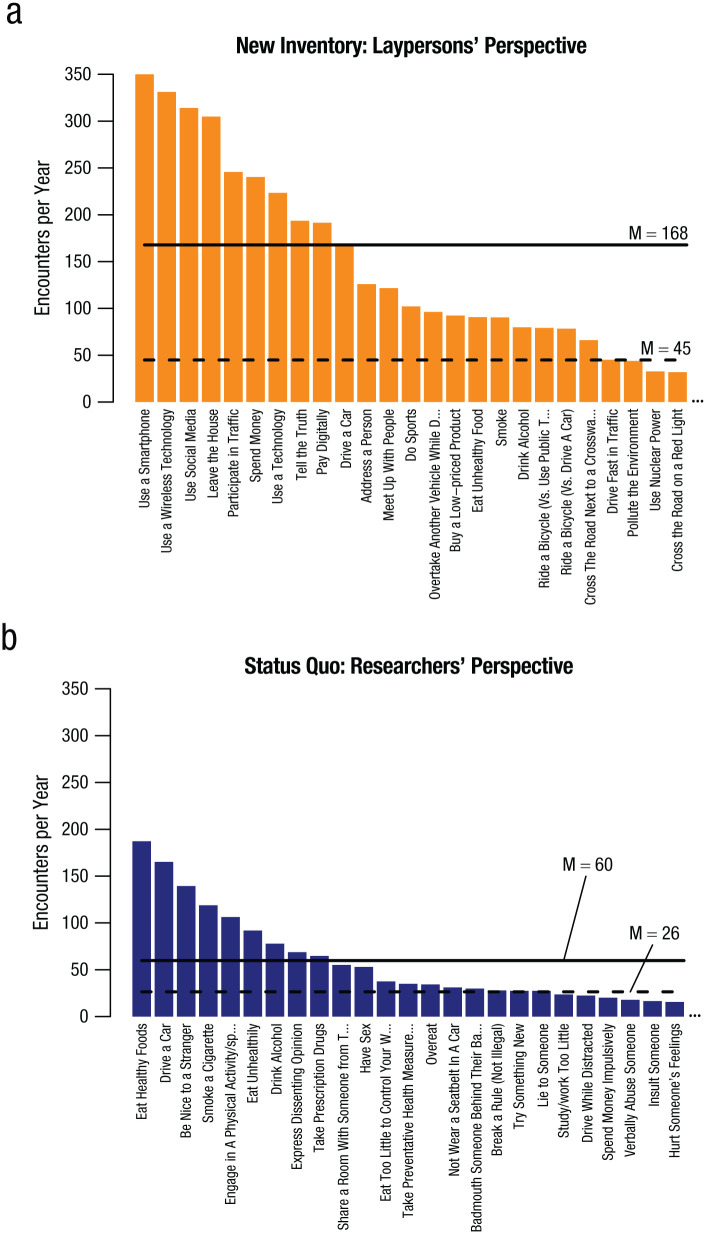
Frequencies of encountering the risky choices per year, separately for the choices of the newly developed inventory (a) and a set of prototypical choices often used in behavioral decision research (b). Solid lines denote the mean frequencies of encountering the 25 most prevalent choices in each inventory; dashed lines denote the mean frequencies across all choices in the respective inventory (i.e., *N* = 100 and *N* = 62, respectively). As a robustness check, we also conducted this analysis with only a subset of the risky choices (see Fig. S10 in the Supplemental Material).

Next we gauged the generalizability of participants’ risk preferences. We did so by investigating how strongly participants’ general risk preferences were associated with whether they chose (or would choose, if they had never encountered a specific choice before) the risky versus the safe option in the 162 risky choices. In doing so, we also compared whether the association between participants’ risk preferences and their choices differed depending on which inventory of risky choices was used as outcome criterion (laypersons’ perspective vs. researchers’ perspective; RQ 4b). Moreover, we compared whether the generalizability of participants’ general risk preferences varied depending on whether they reported their actual choice behavior during past encounters of a risky choice (risky vs. safe option chosen), or—provided that they had never encountered a specific choice before—their hypothetical choice behavior in a future encounter of a risky choice (RQ 4c).

To this end, we implemented a second Bayesian mixed-effects model with the choice behavior (i.e., choice of the risky vs. safe option) as the dependent variable. Independent variables were a participant’s general risk preference, the inventory of risky choices (laypersons’ perspective vs. researchers’ perspective), whether a choice was previously encountered by a participant or not, and the interactions of the latter two variables with a participant’s general risk preference; gender, age, and position of presentation were covariates (see Section II.d in the Supplemental Material for full model specifications). This model suggested that an increase of 1 *SD* in general risk preference was associated with an odds ratio of 1.13 (95% HDI = 1.08, 1.17) of choosing the risky option. Moreover, the odds ratio of choosing the risky option in the choices of the newly developed inventory (i.e., laypersons’ perspective)—relative to in the risky choices currently studied in behavioral decision research (i.e., researchers’ perspective)—was 2.54 (95% HDI = 2.48, 2.61). The odds of choosing the risky option were even more strongly associated with whether or not a participant had previously encountered a specific risky choice, with the former relating to an odds ratio of 12.06 (95% HDI = 11.74, 12.4). Gender, age, and the position of presentation of a risky choice were not associated with any changes in the odds credibly different from 1. The same was true for the interactions between the type of inventory and general risk preference, as well as between whether a choice was previously encountered or not and general risk preference, respectively. All model parameters are reported in Table S5 in the Supplemental Material.

## Discussion

What are the risky choices people are confronted with in our complex and fast-changing world? This is arguably not only an inherently interesting and important question for policymakers and various stakeholders, but is also of fundamental relevance to many areas of the behavioral sciences. For instance, when developing and calibrating novel measurement tools aimed at capturing how people make decisions under risk and uncertainty, it is key to test how well such tools generalize to risky choices people indeed face in real life. To date, such generalizability tests remain scant, and if they are conducted at all, they sometimes rely on seemingly outdated choices that may not be timely and relevant to most people anymore. In this article we have thus developed and analyzed a systematic inventory of timely and relevant risky choices, which has resulted in five key insights.

First, the risky choices that persons from diverse and large population samples consider relevant in modern life can be summarized in a concise inventory of 100 risky choices. This inventory includes both high-stakes choices, such as whether to accept a new job or have children, and also rather mundane choices, such as whether to travel and tell the truth—some of these high-stakes choices were previously also considered *transformative decisions* (Chituc et al., 2021). Although some unique patterns emerged in terms of how often persons of different subgroups reported specific choices, overall there was a remarkable consistency to this inventory. Moreover, a subset of the risky choices appeared similarly in recent investigation of big life decisions (e.g., starting a new job, or getting married; Camilleri, 2023; see also Arslan et al., 2020; Steiner et al., 2021), speaking to the relevance and robustness of the newly developed inventory.

Second, behavioral research often assesses people’s risk preferences separately in different life domains (Blais & Weber, 2006; Dohmen et al., 2011; Highhouse et al., 2016), and the choices of the current ecology of risk span these life domains broadly. Moreover, the high domain homogeneity scores indicated that participants assigned conceptually equivalent choices quite consistently to the same life domain. Finally, the distribution of risky choices to different life domains stayed fairly consistent across different subgroups of the population and across multiple points in time. Together, these findings demonstrate that the commonly studied life domains are useful and adequate even in view of the novel risky choices a fast-changing world entails.

Third, across snapshots of the ecology of risk 1 year apart from each other and under fundamentally different circumstances, very few changes occurred at the aggregate level regarding which risky choices were reported (and in which life domains). Yet individually participants tended to report different choices, suggesting that in itself the ecology of risk remained impressively stable but that individuals are constantly confronted with different choices from this ecology. Future research should evaluate to what extent the apparent robustness and stability of the ecology of risk also persists over even longer periods of time.

Fourth, the independent follow-up assessment (Study 3) revealed that participants had experienced the risky choices of the newly developed inventory almost twice as often as the choices behavioral research targets in the form of frequency measures. This insight implies that the status quo of focusing on risky choices selected by researchers is at least partly outdated, warranting an exploration of whether the newly developed inventory may be better suited for the study of real-life risky choices (e.g., as a benchmark when developing novel measurement instruments; but see also the Limitations section below).

Fifth, an increase of 1 *SD* in a person’s risk preference translated to a 1.13-fold increase in the probability of choosing the risky options in the various choices. The construct of risk preference thus entails a considerable degree of generalizability, at least for mean-level behaviors. Yet to fully understand the often dynamic and context-dependent risky choices of real life, it may be essential to also consider state-specific and situational factors (e.g., Lob et al., 2025).

### Limitations and future research

Although we collected large population samples to increase the robustness of the newly developed inventory, these samples were recruited only in Switzerland—a very safe country with, for instance, a very low crime rate. In addition, although multiple snapshots indicated a high stability of the ecology of risk across time, the overall study period was constrained to approximately 4 years. The newly developed inventory should thus not be directly taken as a measurement scale and used blindly to gauge real-life risk taking in other cultures and populations. Rather, the inventory may serve as a blueprint to evaluate whether and to what extent these (or further) choices are relevant in other contexts and times. More generally, the current approach can be seen as an illustration of how psychological science can and should learn from the experiences of laypersons and perceptions from real life.

Furthermore, our studies intentionally did not include a definition of what constitutes a risky choice, to thus tailor the collection of risky choices from a layperson’s perspective. Consequently, the inventory likely comprises salient choices that laypersons recalled and that they considered risky, which may involve particularly frequent and particularly impactful choices. It is important to note that laypersons and experts may disagree in their conceptualization of the term “risk” (e.g., Holzmeister et al., 2020); this is consistent with the results of Study 3 and with follow-up work comparing these two perspectives on risky choices in more detail (Fischer et al., 2025). Naturally, we cannot evaluate whether a specific framing of a risky choice (e.g., as a choice consisting of options with variable outcomes) would have affected the reported choices; this could be an interesting subject for future research (e.g., Millroth, 2023).

### Final conclusions

In this article we have shed light on the ecology of risk through the eyes of laypersons, to overcome the status quo of the top-down perspective often adopted by researchers of the decision sciences (see Fischer et al., 2025) and to thus contribute to a better understanding of what may be exigent risky choices in everyday life. We do not argue that the laypersons’ perspective should be considered the exclusive ground truth regarding what constitutes relevant risky choices, but we believe that psychological science can and should take into greater account the real-life perspective, to avoid fine-tuning existing theories to potentially obsolete risky choices and to avoid researching outdated phenomena. In this sense, the rich insights from the current, data-driven approach may be informative for policymakers (i.e., who is at risk of being confronted with which risky choices?) and researchers alike (i.e., which risky choices may be good criteria to calibrate novel measurement instruments?).

There have been repeated calls for more discovery-oriented research and a greater emphasis on description in psychological science (e.g., Cooper, 2016; Oberauer & Lewandowsky, 2019; Rozin, 2009). We believe that this article addresses this need and thereby closes an important gap in the field of behavioral decision research. We hope our work can possibly also serve as an illustration of how psychological science could study important real-life phenomena beyond that of risky choices in the future.

## Supplemental Material

sj-pdf-1-pss-10.1177_09567976251384975 – Supplemental material for Mapping the Ecology of Risk: 100 Risky Choices of Modern Life Supplemental material, sj-pdf-1-pss-10.1177_09567976251384975 for Mapping the Ecology of Risk: 100 Risky Choices of Modern Life by Renato Frey and Olivia Fischer in Psychological Science

## Transparency

*Action Editor:* Derek M. Isaacowitz

*Editor:* Simine Vazire

*Author Contributions*

**Renato Frey:** Conceptualization, Data curation, Formal analysis, Funding acquisition, Investigation, Methodology, Project administration, Resources, Software, Supervision, Validation, Visualization, Writing – original draft, Writing – review & editing.

**Olivia Fischer:** Conceptualization, Data curation, Formal analysis, Investigation, Methodology, Project administration, Resources, Validation, Visualization, Writing – original draft, Writing – review & editing.

**Artificial Intelligence:** No artificial-intelligence-assisted technologies were used in this research or the creation of this article.

**Ethics:** This research received approval from the ethics committee of the Faculty of Psychology, University of Basel (Studies 1 and 2; No. 021–19–2) and from the ethics committee of Faculty of Arts and Social Sciences, University of Zurich (Study 3; No. 23–04–12).

**Open Science Framework (OSF):** To facilitate long-term preservation, we registered all OSF files at https://osf.io/gcfz5. Computational reproducibility: The computational reproducibility of the results in the main article (but not the supplementary materials) has been independently confirmed by the journal’s STAR team.

**Open Practices:** Preregistration: The preliminary inventory, the research questions, the methods for the inventory validation (A2), and the analysis plan for Studies 1 and 2 were preregistered at https://osf.io/kbs5u/ (October 12, 2021). There were minor deviations from the analysis plan in how we addressed RQ 3 (see the Supplemental Material, Section III.a). Materials: All study materials are publicly available. The instructions are reported in the Method section, and the materials processed during inventory development are available in the data set. The code base of the study is available at https://osf.io/m9t5v/. Data: All primary data are publicly available at https://osf.io/m9t5v/. All analysis scripts are publicly available at https://osf.io/m9t5v/. For Study 3, prior to data collection, the research questions, methods, and analysis plan were preregistered at https://osf.io/kthpr/ (May 2, 2023). There were minor deviations from the analysis plan in how we addressed RQ 4 (see the Supplemental Material, Section III.b). All study materials are publicly available. The instructions are reported in the Method section, and the items are contained in the data set. The code base of the study is available at https://osf.io/m9t5v/. All primary data are publicly available at https://osf.io/m9t5v/. Analysis scripts: All analysis scripts are publicly available at https://osf.io/m9t5v/.

## Notes

1. By “ecology of risk,” we mean the set of risks that people perceive and potentially engage with in their physical and social environment, akin to the definition of ecology provided by the APA Dictionary of Psychology (American Psychological Association, 2025).
