# Supplementary material for: Mapping the Ecology of Risk: 100 Risky Choices of Modern Life
Source: Psychol Sci. 2025 Nov 6;36(11):846–61. doi: 10.1177/09567976251384975 (PMC13080786; doi:10.1177/09567976251384975)
Supplement: Supplementary file 1 [file sj-pdf-1-pss-10.1177_09567976251384975.pdf]

Supplemental Materials

Mapping the ecology of risk: 100 risky choices of modern life

Renato Frey & Olivia Fischer

University of Zurich

August 27, 2025

Contents

I Detailed methods 3

I.a Collection of risky choices (original German wording) 3

II Extended results 3

II.a Robustness checks 3

II.b RQ 2: Distribution of life domains as a function of the level of analysis 4

II.c RQ 3: Shifts from before to during the Covid-19 pandemic 4

II.d RQ 4: Prevalence of risky choices and generalizability of people's risk preferences 4

III Deviations from the preregistered analysis plan 5

III.a Analyses addressing RQ 3 5

III.b Analyses addressing RQ 4 5

IV Figures 7

V Tables 22

I Detailed methods

I.a Collection of risky choices (original German wording)

> Menschen unterscheiden sich sehr, wie sie mit den Chancen und Risiken des modernen Lebens umgehen.
>
> [Personal perspective]: Versuchen Sie sich jetzt bitte an **eine wichtige Entscheidung aus Ihrem Leben zu erinnern**, wo Sie die Wahl hatten, ein Risiko einzugehen oder dieses zu vermeiden – und Sie sich schlussendlich **für [gegen] das Risiko** entschieden haben.
>
> [Other perspective]: Überlegen Sie sich jetzt bitte **eine wichtige Entscheidung, die viele Leute treffen müssen**, und bei der die Leute die Wahl haben, ein Risiko einzugehen oder dieses zu vermeiden. Denken Sie dabei bitte an eine Entscheidung, wo sich Ihrer Ansicht nach die meisten Leute schlussendlich **für [gegen] das Risiko entscheiden**.
>
> Ich musste mich entscheiden, ob ich ein Risiko eingehe und [risky option] oder ob ich ein Risiko vermeide und deshalb [safe option].
>
> Bei dieser Entscheidung habe ich mich schlussendlich **für das Risiko** entschieden, nämlich dass ich [risky option].

II Extended results

II.a Robustness checks

As preregistered, we compared the distributions of risky choices and their associated life domains between participants of the cross-sectional sample and the robustness-check sample. In contrast to the former, the latter were not recruited in the context of another study on people's risk perceptions of 5G (see materials and procedures section in main text). This robustness check thus enabled gauging any potential influences and carry-over effects. To compare the respective proportions and counts of choices and domains, we conducted Bayesian proportion tests and correlation analyses using the R package BayesFactor (Morey & Rouder, 2018).

Level of choices

We first compared the top 10 risky choices of each sample, which together included 12 unique choices (Figure S11, panel A). Comparing the proportions of these 12 top choices in the two samples with Bayesian proportion tests revealed no credible differences in nine cases (i.e., Bayes Factors clearly smaller than 1) and credible differences in three cases (i.e., Bayes Factors clearly larger than 1; all Bayes Factors are depicted in Figure S11). Next, we gauged the consistency between the two samples across all 100 inventory choices, by estimating Bayesian Spearman rank correlations. Specifically, we used non-informative priors (i.e., $Beta(1, 1)$) and ran the model with 100,000 iterations. This analysis revealed a moderate correlation of .51 (95% HDI = .36, .64) between the frequencies in the two samples.

Level of life domains

Next, we compared the proportions of the choices' life domains in the cross-sectional and robustness check samples (Figure S11, panel B). In the cross-sectional sample, 118 (27%) choices occurred in the occupational domain, 105 (24%) in the health domain, 64 (15%) in the financial domain, 58 (14%) in the traffic domain, 46 (11%) in the recreational domain, and 41 (10%) in the social domain. In the robustness-check sample, 266 (38%) choices occurred in the occupational domain, 87 (13%) in the health domain, 133 (19%) in the financial domain, 65 (9%) in the traffic domain, 52 (8%) in the recreational domain, and 98 (14%) in the social domain. Bayesian proportion tests revealed no credible differences between the two samples in the proportions of the risky choices in the financial, traffic, recreational, and social domains, but credible differences in the occupational and health domains (see Figure S11 for the respective Bayes Factors).

Summary

The results on the level of domains largely corroborate the results on the level of choices: Both sets of results indicate that participants of the cross-sectional sample and participants of the robustness-check sample by and large reported the same choices and also a comparable number of choices in the various life domains—although some minor divergences did emerge (e.g., somewhat fewer risky choices in the health domain were reported by participants of the robustness-check sample relative to participants of the cross-sectional sample).

II.b RQ 2: Distribution of life domains as a function of the level of analysis

As preregistered, we ran a sanity check comparing the proportions of life domains at the level of inventory choices (as reported in the main text; Figure S6, first data series) with the proportions of life domains at the level of choices originally reported by participants (Figure S6, second data series). The respective proportions were very similar: Of all participant choices, 692 (28%) occurred in the occupational domain, 429 (18%) occurred in the health domain, 520 (21%) occurred in the financial domain, 333 (14%) occurred in the social domain, 236 (10%) occurred in the traffic domain, and 228 (9%) occurred in the recreational domain.

To gauge any potential differences between the two levels of analysis, we implemented the same type of Bayesian proportion tests as introduced in section II.a. These tests indicated that the proportions of life domains did not credibly vary as a function of the level of analysis for the occupational, health, social, traffic, and recreational domains, but they varied for the financial domain (see Figure S6 for all Bayes Factors).

II.c RQ 3: Shifts from before to during the Covid-19 pandemic

Level of choices

To examine any shifts in the reported risky choices from before to during the Covid-19 pandemic, we compared the proportions of the top choices in the baseline, cross-sectional, and longitudinal samples (Figure S9). To do so we used the same type of Bayesian proportion tests as introduced in section II.a. The Bayes Factors of these tests indicated that there were no credible differences in the proportions from before to during the pandemic in nine out of the respective top 11 choices when comparing the baseline to both the cross-sectional as well as the longitudinal sample (comprising 13 unique choices in total). In contrast, the baseline sample differed from the cross-sectional (get vaccinated; meet up with people) and longitudinal (meet up with people; leave the house) samples in the reported proportions of two choices each. In these cases, relatively more people reported these choices in the two samples collected during the Covid-19 pandemic.

II.d RQ 4: Prevalence of risky choices and generalizability of people's risk preferences

To address RQs 4a–c we ran two Bayesian mixed-effects models using the R package brms (Bürkner, 2017). We ran each model with 4 chains and 3,000 iterations per chain (i.e., 1,500 warmup and 1,500 post-warmup). As preregistered, we employed weakly informative priors for the fixed effects, specifically $\mathcal{N}(0, 10)$. Furthermore, we used brms' default priors for the random intercepts. In the zero-inflated negative binomial model to estimate the frequency of encountering the various risky choices (RQ 4a), we used brms' default priors for the shape (overdispersion) and zero-inflation parameters. The models converged well, with all R-hat values at 1.

In what follows we report the full results regarding the first mixed-effects model beyond the fixed effects that are reported in the main text. Specifically, the shape and zero-inflation parameters describe the degree of overdispersion and zero-inflation, respectively. There was a moderate degree of overdispersion (0.2; 95% HDI = 0.19, 0.21) and substantial zero-inflation (.46; 95% HDI = .45, .47). That is, the zero-inflation parameter indicates that an estimated 46% of the observations on choices typically used in behavioral decision research included excess zeros that the negative binomial component alone could not account for, keeping all other variables constant.

III Deviations from the preregistered analysis plan

III.a Analyses addressing RQ 3

RQ 3a: Number of top risky choices

Both in the case of identifying the most commonly reported risky choices in the baseline and cross-sectional samples as well as in the baseline and longitudinal samples, 11 instead of the preregistered 10 top choices were identified using the preregistered approach, as several choices were reported equally frequently and were thus tied in their position. The results reported in the main text represent the union of these two sets of choices, the proportions of which are also depicted in S9. Similarly, in the robustness check analyses 12 instead of 10 top choices were identified.

RQ 3a: Alluvial plot of change for the top choices

We did not create an alluvial plot of change to illustrate any changes in the most commonly reported risky choices from the baseline to the longitudinal samples as we had preregistered. Given that only very few participants reported one of those top choices both in the baseline and longitudinal sample, the alluvial plot on the level of these top choices would only illustrate the change for this small subset of participants, which could be misconstrued as being representative for the two samples as a whole.

RQ 3b: Bayesian correlations on the level of domains

Unlike what we had preregistered, we did not conduct Bayesian correlation analyses of the frequency of the risky choices' life domains in the different samples (both for RQ 3 and the robustness checks). Due to the small number of domains, these correlations would have resulted in relatively wide 95% HDIs, rendering this analysis uninformative.

III.b Analyses addressing RQ 4

RQ 4: Number of risky choices

In our preregistration of study 3 (https://osf.io/kthpr/) we specified that participants would rate the frequencies of having encountered 164 risky choices, namely, 102 stemming from the inventory representing laypersons' perspective, and 62 stemming from the inventory representing researchers' perspective. However, at the time of submitting the preregistration for study 3 we had not yet applied the last of our criteria that we preregistered for studies 1 and 2 (i.e., to remove those inventory choices with the lowest classification confidence, to thus arrive at 100 inventory choices). For this reason, we only used data from 162 risky choices (i.e., 100 choices of the inventory newly developed in studies 1 and 2, plus 62 risky choices from the research-perspective inventory) in order to address RQs 4a–c.

RQ 4: Removal of extreme outliers

Upon inspection of the data, we noticed that some participants reported excessively higher frequency ratings compared to the others. Specifically, 59 participants reported a mean frequency (i.e., across all risky choices) that was at least 10 times higher than the median across all participants (we aimed to choose a relatively conservative threshold yet the threshold itself is arbitrary). We decided to remove the data of these participants, which resulted in the final sample size of 933.

RQ 4: Model specifications

We anticipated that participants' self-reported frequencies of having encountered the various risky choices (i.e., RQ 4a) would follow a Poisson distribution. However, due to overdispersion in the count data (i.e., the variance was larger than the mean) and zero-inflation (i.e., a larger proportion of zeros than would be expected from a Poisson distribution), we fit a zero-inflated negative binomial (Yau et al., 2003). This model can deal with both features of the data that violate the assumptions of a Poisson distribution and was therefore more adequate for the data.

Moreover, RQs 4b and 4c were intended to investigate whether the associations between a person's risk preference and their inclination to choose risky options depended on the type of inventory used, and on whether choices were actually encountered in the past versus imagined to occur in the future. In other words, these RQs aimed to test interactions, yet due to a mistake in the notation of the models we only specified main effects in our preregistration. We corrected this mistake by implementing interaction terms between risk preference and type of inventory (RQ 4b) and between risk preference and whether people experienced a choice in the past (RQ 4c). To accommodate these model specifications, we simplified the random effects structure of the respective preregistered models by only estimating random intercepts for participants.

Finally, for the model estimation process we also removed the data of four participants (i.e., 0.4% of all cases) who reported "non-binary" as their gender; due to the few data points for this level the respective fixed effects cannot be estimated reliably.

IV Figures

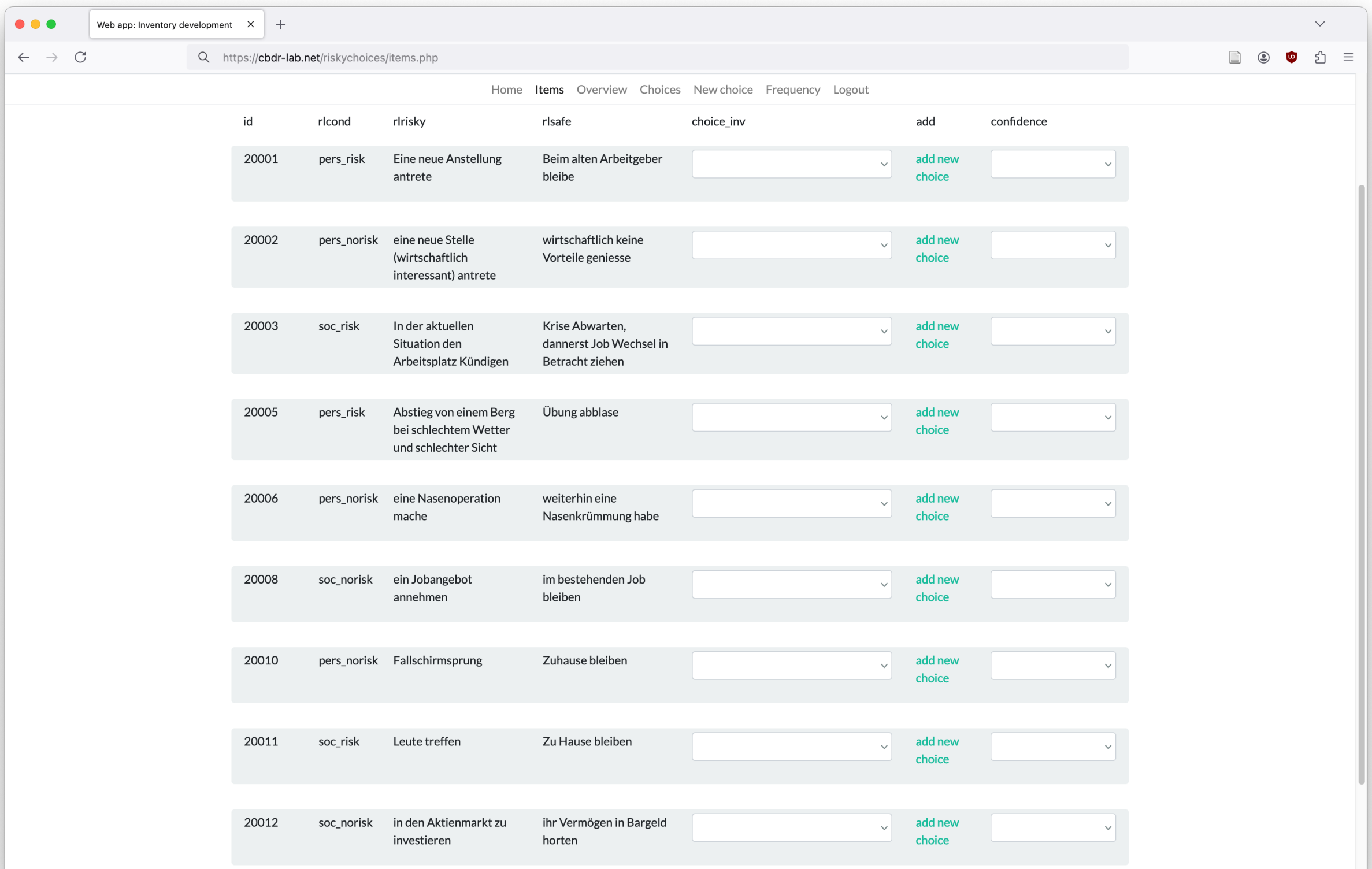

Figure S1: Screenshot of the dedicated web application that we developed and used to streamline the inventory-development process. The current page illustrates the initial classification step during the discovery phase (stage A1), during which it was possible to either assign a participant choice to one of the existing inventory choices, or—provided that none of the latter were suitable to classify a participant choice—to create a new inventory choice.

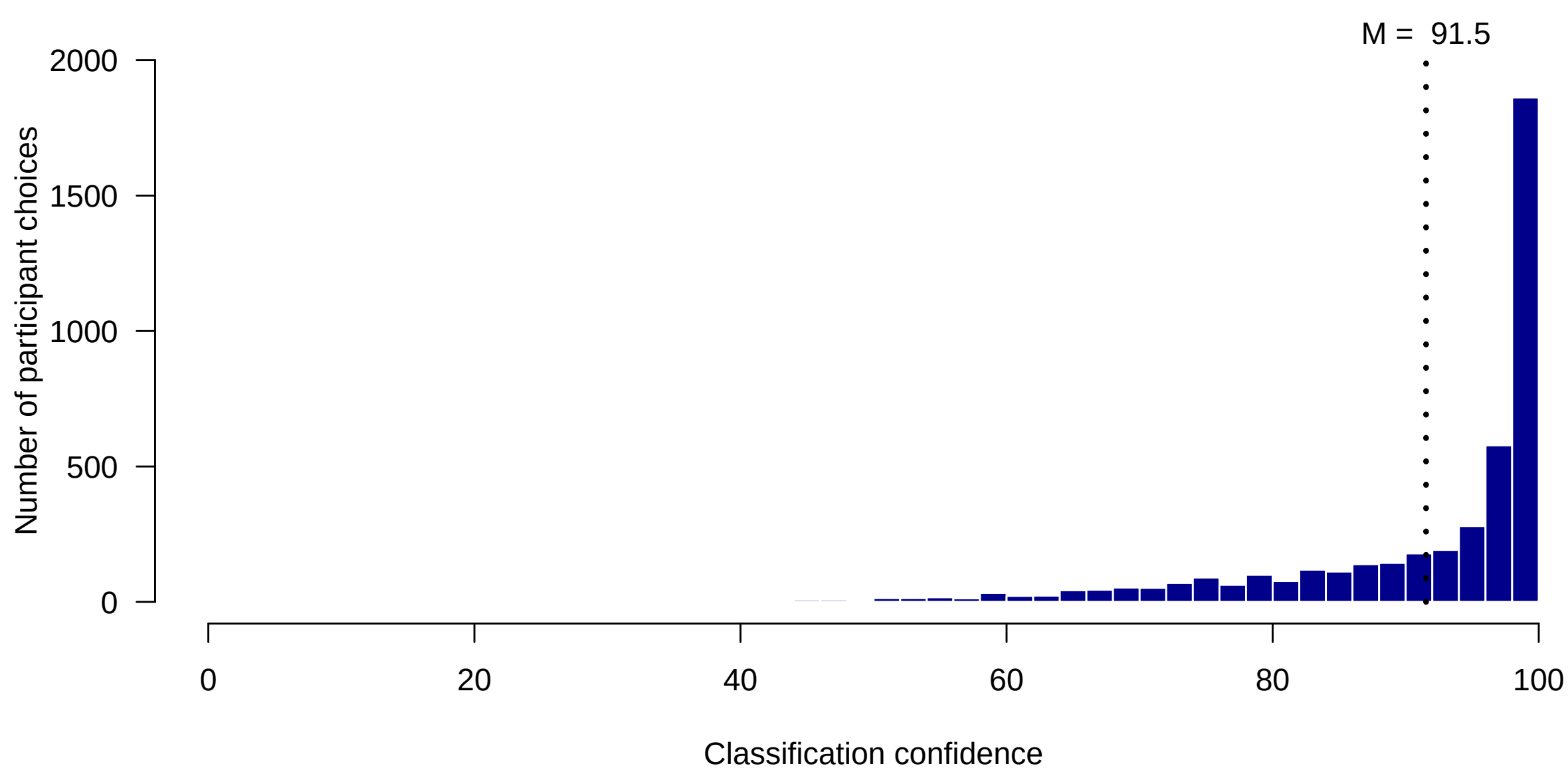

Figure S2: Distribution of classification confidences during the validation phase (stage A2). The classification confidence refers to the average confidence with which the two (out of two, or in case of disagreement, out of three) independent raters assigned each of the 4,380 participant choices to an inventory choice.

#	Risky option	Female	Male	Mean Age	Modal domain	Occup., Fin., Health, Traffic, Social, Recr.
26	Get a certain education or training	14	9	27	Occupational (96%)	22
27	Buy a car	10	11	40	Financial (95%)	20
28	Start a romantic relationship	12	8	39	Social (100%)	20
29	Go on a hike	6	14	51	Recreational (75%)	15, 4
30	Skydive	10	10	45	Recreational (85%)	17, 3
31	Drive fast in traffic	8	12	38	Traffic (85%)	17
32	Take the more challenging path on a hike	5	14	54.8	Recreational (84%)	16, 2
33	Accept a new challenge at work	8	9	51.8	Occupational (88%)	15
34	Get divorced	14	1	54.8	Social (73%)	11, 2, 2
35	Build a house	5	10	48.5	Financial (93%)	14
36	End a romantic relationship	10	4	40	Social (79%)	11, 2, 1
37	Cross the road on a red light	5	9	32	Traffic (79%)	11, 2, 1
38	Accept a job in another industry	5	9	51.3	Occupational (71%)	10, 2, 2
39	Help a person in need	6	7	36	Social (69%)	9, 2, 2
40	Overtake another vehicle while driving one's car	4	9	41	Traffic (85%)	11, 1, 1
41	Do a stay abroad during one's education or training	7	6	32	Occupational (54%)	7, 5, 1
42	Bet money on a gamble	4	9	38	Financial (62%)	8, 4, 1
43	Take an exam	5	7	25	Occupational (100%)	12
44	Do sports	6	6	46.6	Recreational (67%)	8, 4
45	Do a challenging sporting activity	5	7	47.3	Recreational (58%)	7, 4, 1
46	Use a smartphone	8	4	50.1	Social (75%)	9, 1, 1, 1
47	Travel alone	7	4	48.7	Recreational (45%)	5, 4, 1, 1
48	Ski	6	5	44	Recreational (73%)	8, 3
49	Drive a motorcycle	4	7	50.5	Health (36%)	4, 4, 3
50	Drop out of an educational or training program	6	5	42	Occupational (91%)	10, 1

Figure S3: The ecology of risk (continued): Inventory choices 26–50.

#	Risky option	Female	Male	Mean Age	Modal domain	Occup. | Fin. | Health | Traffic | Social | Recr.
51	Ride off-piste	5	6	53.1	Recreational (82%)	9 1 1
52	Carry a pregnancy full term	8	2	45.5	Social (40%)	4 3 2 1
53	Spend money	5	5	38	Financial (90%)	9 1
54	Paraglide	4	6	56	Recreational (100%)	10
55	Dodge the fare in public transportation	6	3	26	Traffic (56%)	5 2 1 1
56	Go to school	4	5	29	Occupational (78%)	7 2
57	Use nuclear power	3	6	58	Health (56%)	5 3 1
58	Drink alcohol	3	6	41	Health (67%)	6 2 1
59	Take out a loan	5	4	38	Financial (89%)	8 1
60	Bungee jump	4	5	39	Recreational (89%)	8 1
61	Take over a business	2	7	59	Occupational (56%)	5 4
62	Disregard safety measures in traffic	5	4	33	Traffic (89%)	8 1
63	Disregard safety measures while riding a bicycle	6	3	38	Traffic (89%)	8 1
64	Change one's education or training	5	3	36	Occupational (100%)	8
65	Get an education or training abroad	3	4	37	Occupational (71%)	5 1 1
66	Travel to a politically unstable region	7	0	50.9	Recreational (43%)	3 3 1
67	Ride a bicycle (vs. use public transportation)	4	3	48.7	Traffic (86%)	6 1
68	Cheat on one's partner	4	3	41	Social (100%)	7
69	Pollute the environment	4	2	50.2	Social (67%)	4 2
70	Cross the road next to a crosswalk	4	2	40	Traffic (83%)	5 1
71	Have unprotected sex	0	6	27	Health (83%)	5 1
72	Tell the truth	3	3	36	Social (67%)	4 1 1
73	Have insurance with a high patient contribution	1	5	43	Financial (67%)	4 2
74	Run a red light	2	4	36	Traffic (100%)	6
75	Get paid one's pension as a lump sum after retirement	1	5	48.8	Financial (100%)	6

Figure S4: The ecology of risk (continued): Inventory choices 51–75.

#	Risky option	Female	Male	Mean Age	Modal domain	Occup. | Fin. | Health | Traffic | Social | Recr.
76	Not get prenatal diagnostics during pregnancy	4	1	50.8	Health (80%)	4, 1
77	Pay digitally	3	2	43	Financial (100%)	5
78	Use a wireless technology	1	4	50.2	Health (60%)	3, 2
79	Eat unhealthy food	1	4	39	Health (100%)	5
80	Go climbing	3	2	40	Recreational (100%)	5
81	Drive a car without a seatbelt	3	2	32	Traffic (100%)	5
82	Use social media	3	1	40	Social (100%)	4
83	Jump off a springboard	1	3	37	Recreational (75%)	3, 1
84	Jump off a bridge	2	2	38	Recreational (100%)	4
85	Undergo chemotherapy	4	0	60	Health (100%)	4
86	Work for a low salary	3	1	46.2	Occupational (50%)	2, 2
87	Live close to 5G antennae	0	4	45.5	Health (75%)	3, 1
88	Give birth naturally	3	1	39	Health (100%)	4
89	Retire early	1	3	60	Financial (50%)	2, 1, 1
90	Remain abroad	1	3	43	Occupational (75%)	3, 1
91	Travel without vaccination	3	0	32	Health (67%)	2, 1
92	Buy a low-priced product	3	0	30	Financial (100%)	3
93	Travel to a different continent	1	2	57	Health (33%)	1, 1, 1
94	Drive without winter tires in the winter	3	0	25	Traffic (100%)	3
95	Demand a pay raise	0	3	33	Financial (67%)	2, 1
96	Address a person	0	3	21	Social (100%)	3
97	Consume drugs	2	1	38	Health (67%)	2, 1
98	Raise a complaint with one's superior	1	2	34	Occupational (100%)	3
99	Ride a bicycle (vs. drive a car)	2	1	47.3	Health (67%)	2, 1
100	Work part-time	1	1	62	Health (100%)	2

Figure S5: The ecology of risk (continued): Inventory choices 75–100.

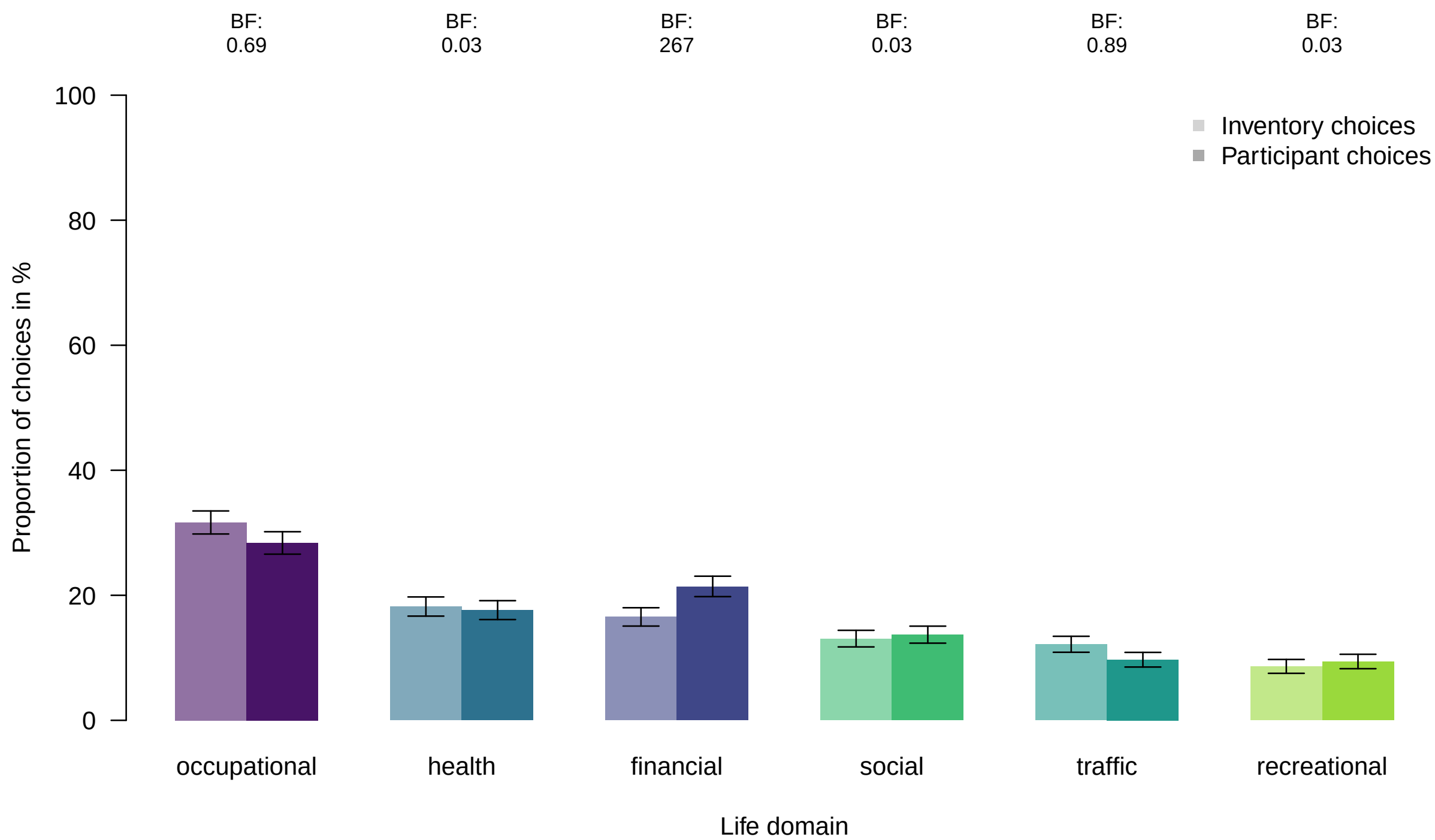

Figure S6: Proportions of life domains for two levels of analysis (see section II.b). The first data series depicts the proportions of life domains as determined based on the modal domains of the inventory choices (i.e., counting how many participant choices were assigned to an inventory choice). The second data series depicts the proportions of life domains as determined by directly counting the life domains of the original participant choices. The error bars represent 95% HDIs of the estimated proportions. BF = Bayes Factor indicating the evidence that two proportions are credibly different from each other (values smaller than 1 indicate that there is no difference).

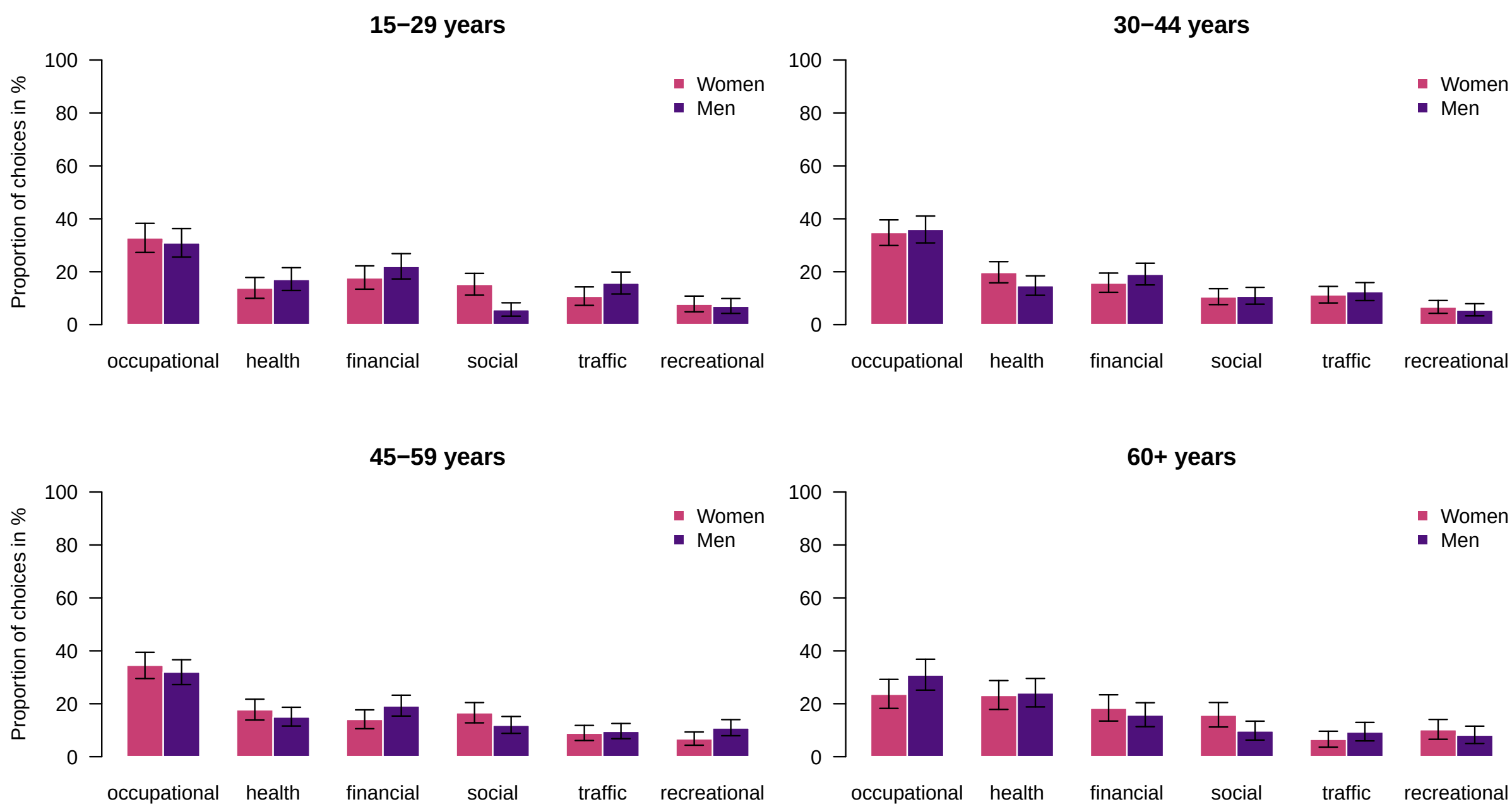

Figure S7: Proportion of risky choices occurring in different life domains, separately for four age groups and gender. The error bars represent 95% HDIs of the estimated proportions.

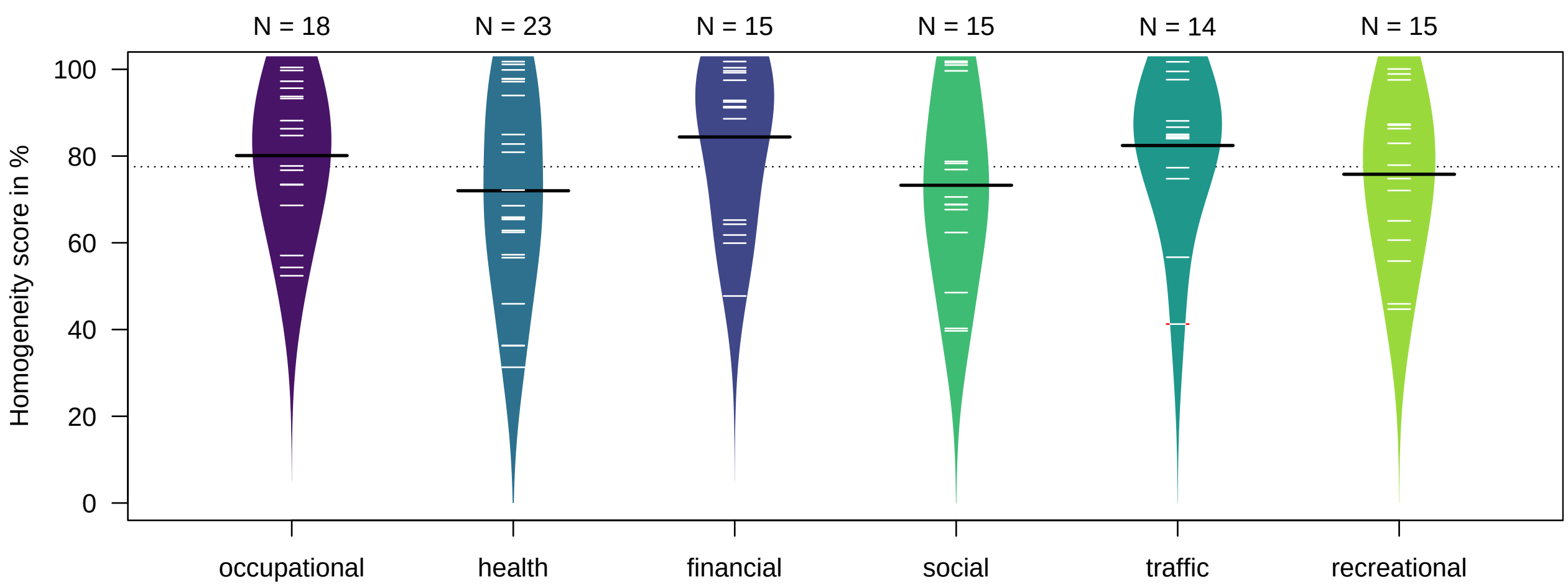

Figure S8: Domain homogeneity scores of the inventory choices, grouped by the modal life domain of each inventory choice. A homogeneity score of 100% indicates that all participant choices assigned to this inventory choice were classified as the same life domain.

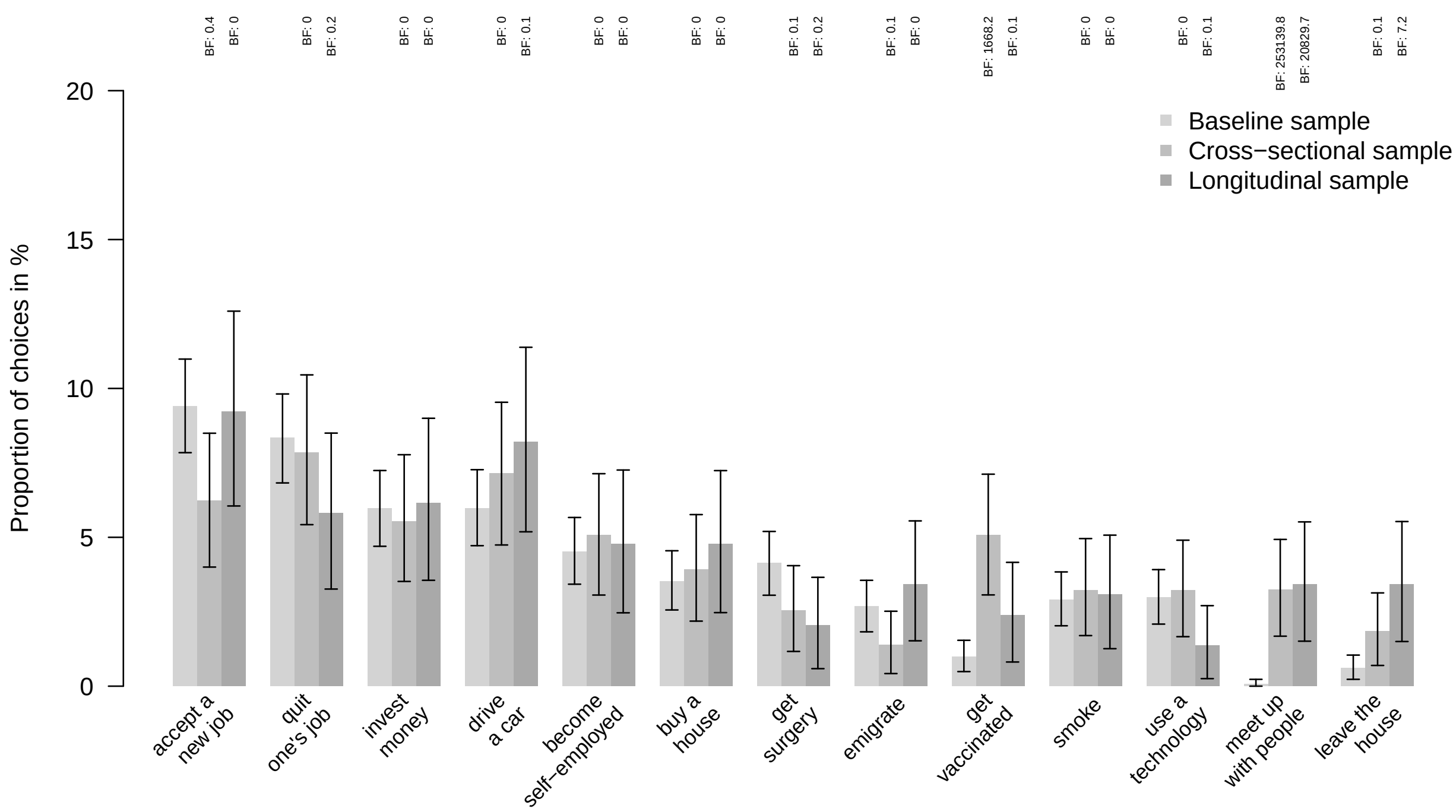

Figure S9: Proportions of the most frequently reported risky choices in different samples, both before the Covid-19 pandemic (baseline sample) and during the pandemic (cross-sectional sample, longitudinal sample; see section II.c). The error bars represent 95% HDIs of the estimated proportions. BF = Bayes Factor indicating the evidence that the shown proportion differs from the equivalent proportion in the baseline sample (values smaller than 1 indicate that there is no difference).

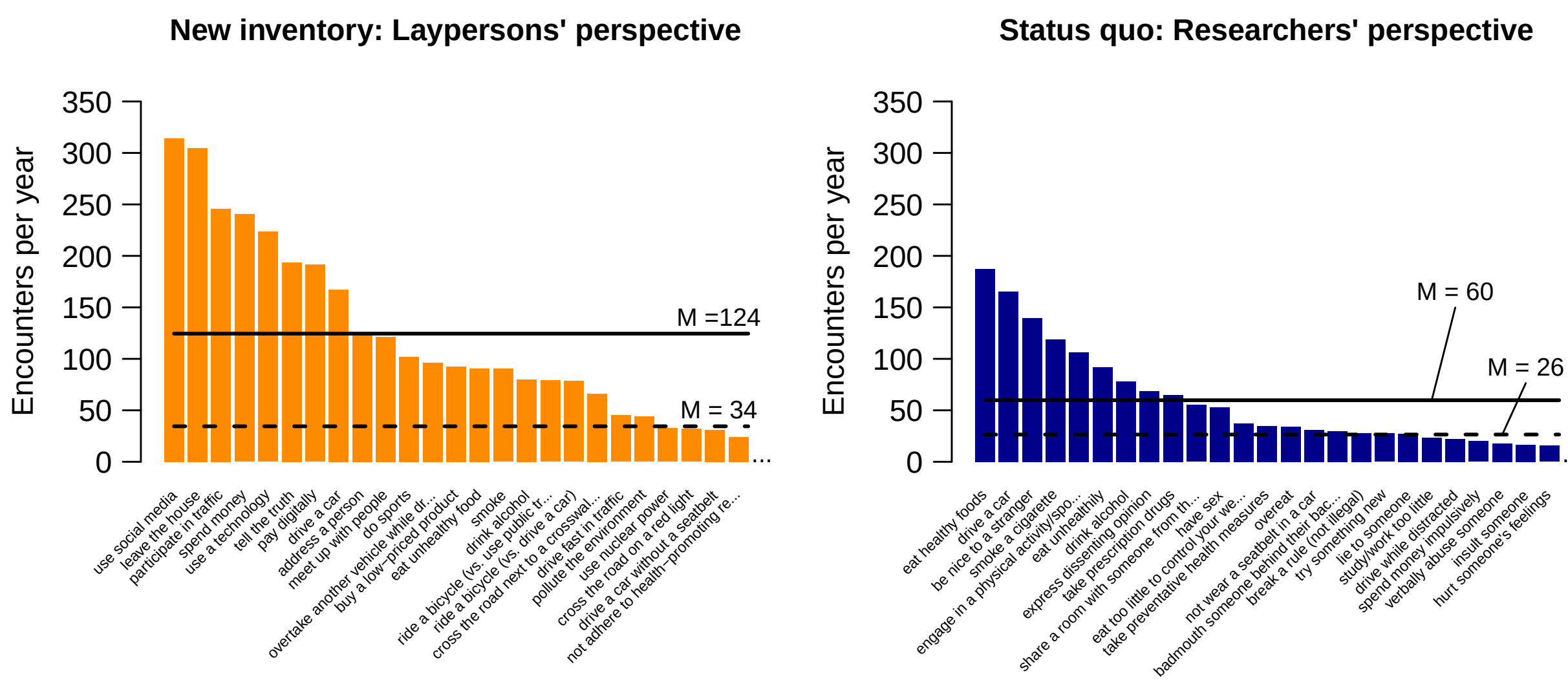

Figure S10: Robustness check: Frequencies of encountering the risky choices per year, separately for the newly developed inventory and a set of prototypical choices often used in behavioral decision research. In this robustness check, the three choices highlighted in table S6 were removed (i.e., choices that could have been triggered only in the samples in which participants were also prompted to report their risk perceptions of 5G).

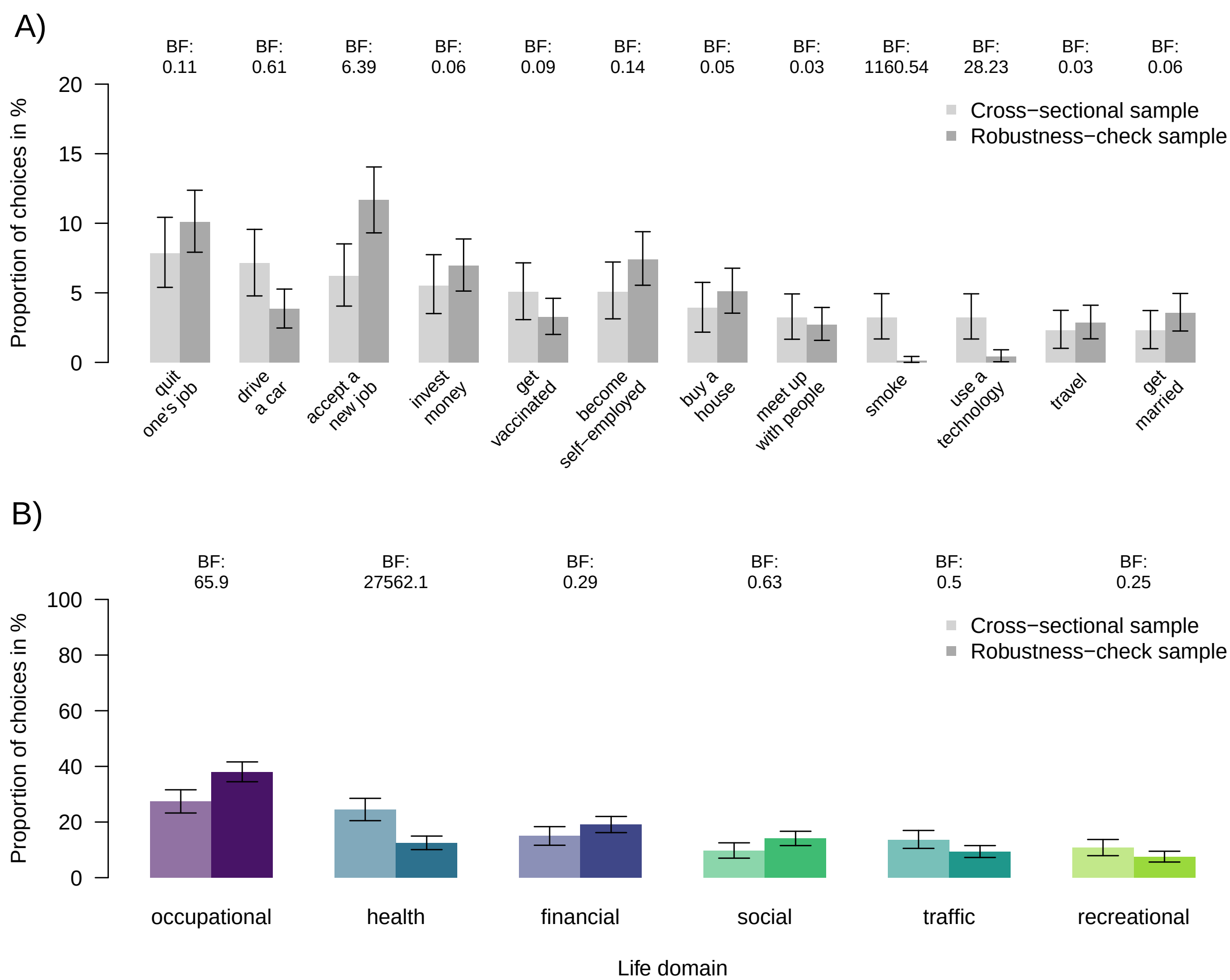

Figure S11: Robustness check: Proportions of the most frequently reported risky choices (panel A) and their life domains (panel B) reported in the cross-sectional and robustness-check samples (see section II.a). The error bars represent 95% HDIs of the estimated proportions. BF = Bayes Factor indicating the evidence that two proportions are credibly different from each other (values smaller than 1 indicate that there is no difference).

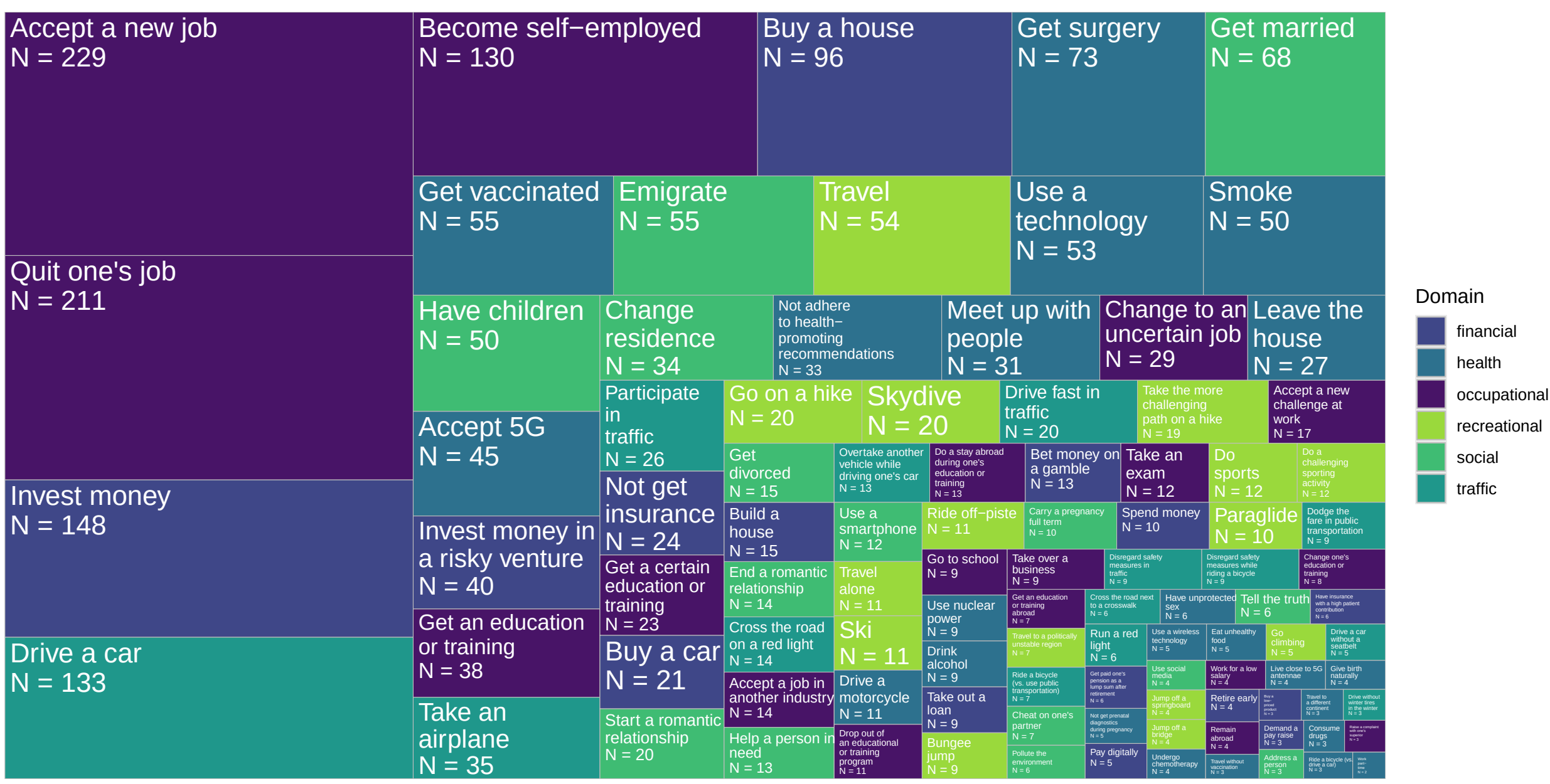

Figure S12: Treemap figure of the ecology of risk.

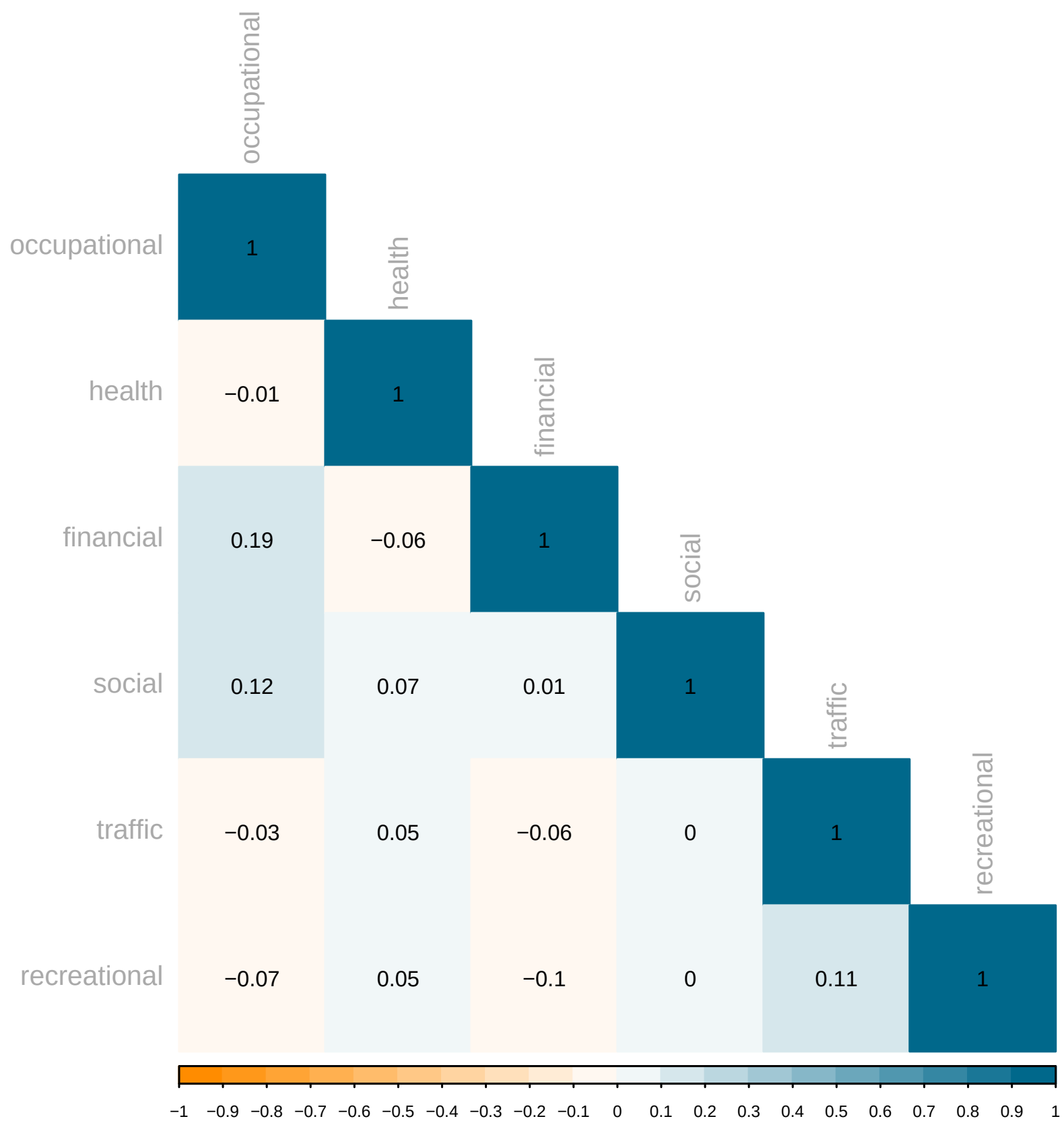

Figure S13: Co-occurrence of life domains, as measured with the correlations between the domain profiles of the 100 inventory choices (i.e., the counts of the self-reported domains of all participant choices assigned to an inventory choice).

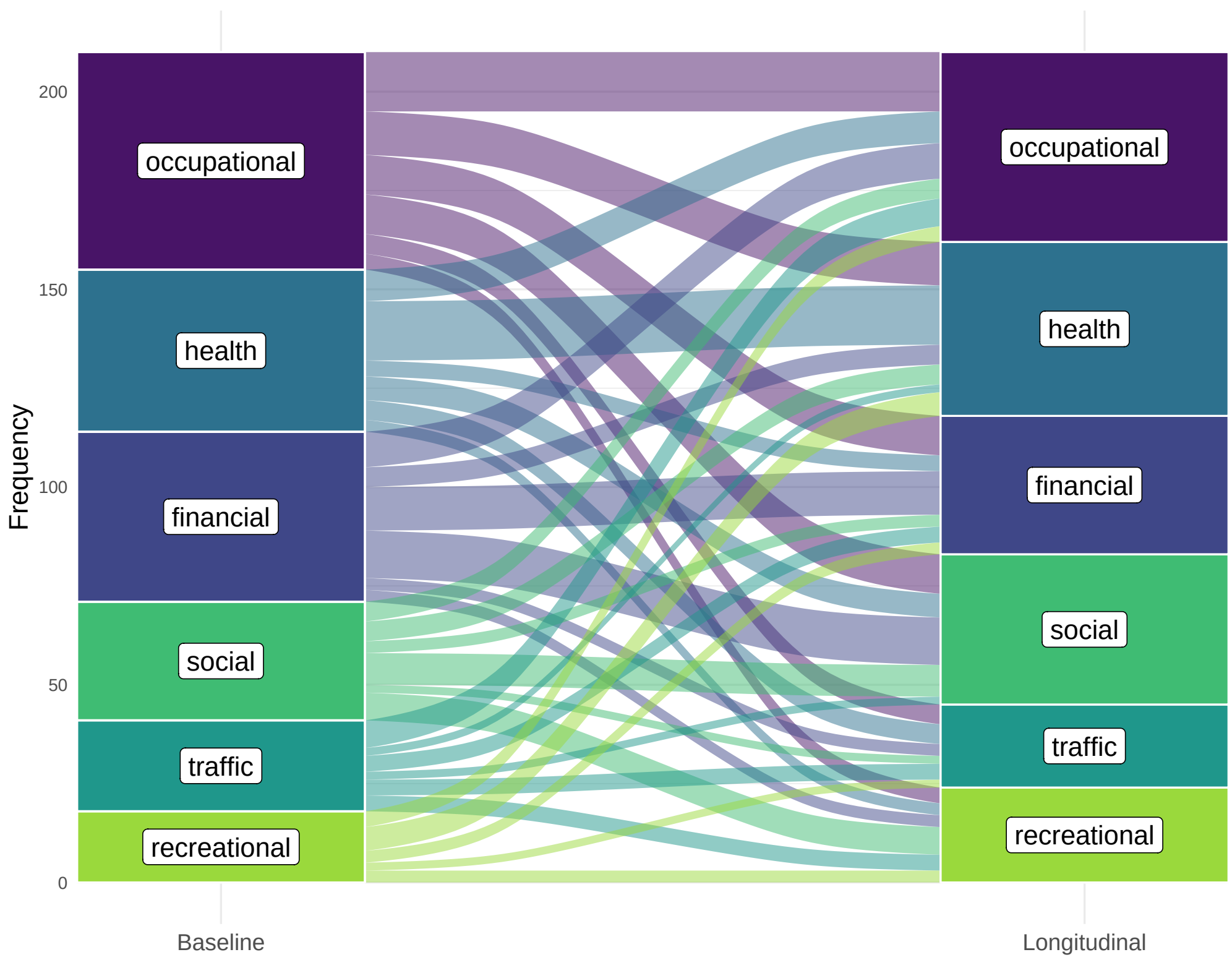

Figure S14: Alluvial plot showing the intraindividual stability versus change of the life domains that participants reported across the two points in time.

V Tables

Table S1: **Overview of samples and participant characteristics.**

Study		1 (Nov. 2019)	2 (Nov. 2020)	2 (Nov. 2020)	2 (Nov. 2020)	3 (May 2023)
Sample		Baseline	Cross-sectional	Longitudinal	Robustness-check	Follow-up
N		2178	718	448	1036	933
Gender	female	1066 / 48.9%	362 / 50.4%	181 / 40.4%	511 / 49.3%	469 / 50.3%
	male	1112 / 51.1%	356 / 49.6%	267 / 59.6%	525 / 50.7%	460 / 49.3%
Age		44.7 (SD=16.19)	45.25 (SD=16.02)	47.52 (SD=15.75)	45.39 (SD=16.14)	46.37 (SD=16)
Edu.	basic education	828 / 38%	257 / 35.8%	149 / 33.3%	378 / 36.5%	334 / 35.8%
	high school	493 / 22.6%	163 / 22.7%	96 / 21.4%	218 / 21%	193 / 20.7%
	college degree	837 / 38.4%	292 / 40.7%	201 / 44.9%	431 / 41.6%	405 / 43.4%
	none	20 / 0.9%	6 / 0.8%	2 / 0.4%	9 / 0.9%	1 / 0.1%
Occ.	in education	188 / 8.6%	65 / 9.1%	22 / 4.9%	83 / 8%	64 / 6.9%
	employed	1401 / 64.4%	474 / 66%	296 / 66.1%	655 / 63.2%	618 / 66.2%
	self-employed	140 / 6.4%	28 / 3.9%	21 / 4.7%	68 / 6.6%	51 / 5.5%
	house-wm./man	110 / 5.1%	39 / 5.4%	17 / 3.8%	53 / 5.1%	32 / 3.4%
	retired	306 / 14.1%	98 / 13.6%	87 / 19.4%	148 / 14.3%	151 / 16.2%
	none	32 / 1.5%	14 / 1.9%	5 / 1.1%	29 / 2.8%	17 / 1.8%

Table S2: Most frequently reported risky choices among women, separately for four age groups.

#	Risky option	Safe option	Count
15–29 years			
1	quit one's job	not quit one's job	26
2	accept a new job	not accept the job	18
3	invest money	not invest money	15
4	get a certain education or training	get another education or training	11
5	emigrate	not emigrate	10
30–44 years			
1	quit one's job	not quit one's job	43
2	accept a new job	not accept the job	32
3	drive a car	not drive a car	21
4	get married	not get married	19
5	buy a house	not buy a house	17
45–59 years			
1	invest money	not invest money	29
2	drive a car	not drive a car	19
3	quit one's job	not quit one's job	19
4	get an education or training	not get an education or training	10
5	accept a new job	not accept the job	9
60+ years			
1	accept a new job	not accept the job	40
2	quit one's job	not quit one's job	23
3	buy a house	not buy a house	22
4	become self-employed	stay employed	22
5	get married	not get married	18

Table S3: Most frequently reported risky choices among men, separately for four age groups.

#	Risky option	Safe option	Count
15–29 years			
1	quit one's job	not quit one's job	52
2	accept a new job	not accept the job	32
3	drive a car	not drive a car	26
4	buy a house	not buy a house	21
5	become self-employed	stay employed	21
30–44 years			
1	accept a new job	not accept the job	27
2	get surgery	not get surgery	22
3	invest money	not invest money	15
4	travel	not travel	10
5	drive a car	not drive a car	10
45–59 years			
1	accept a new job	not accept the job	50
2	invest money	not invest money	31
3	quit one's job	not quit one's job	28
4	drive a car	not drive a car	19
5	become self-employed	stay employed	19
60+ years			
1	become self-employed	stay employed	26
2	accept a new job	not accept the job	21
3	drive a car	not drive a car	14
4	quit one's job	not quit one's job	14
5	accept 5G	not accept 5G	14

Table S4: Estimated model parameters (RQ 4a).

Parameter	Frequency (95% HDI)
Intercept	49.4 (45, 54.3)
Perspective (laypersons)	33.5 (27.9, 40)
Gender (male)	1.3 (-4.2, 8.9)
Age	-11.1 (-12.2, -9.7)
Position of presentation	0 (0, 0)

Table S5: Estimated model parameters (RQs 4b and 4c).

Parameter	Odds (95% HDI)
Risk preference	1.13 (1.08, 1.17)
Perspective (laypersons)	2.54 (2.48, 2.61)
Choice encountered (yes)	12.06 (11.74, 12.4)
Gender (male)	1.05 (0.98, 1.13)
Age	0.98 (0.95, 1.02)
Position of presentation	1 (1, 1)
Risk preference * Inventory (layp.)	1.02 (0.99, 1.04)
Risk preference * Choice enc. (yes)	0.99 (0.96, 1.02)

Table S6: List of inventory choices that were reported in the cross-sectional sample but not in the robustness-check sample (N = 17). Choices potentially related to the questions on 5G risk perceptions (i.e., in the cross-sectional sample) are highlighted.

398	smoke	not smoke	13	0
**292**	**accept 5G**	**not accept 5G**	**11**	**0**
1221	do a challenging sporting activity	do an easier sporting activity	2	0
**414**	**use a smartphone**	**not use a smartphone**	**3**	**0**
1239	travel to a politically unstable region	travel to a politically stable region	2	0
1230	ride a bicycle	use public transportation	1	0
1213	pay digitally	choose a more traditional method of paying	1	0
**1212**	**use a wireless technology**	**use a wired technology**	**2**	**0**
379	go climbing	not go climbing	1	0
315	drive a car without a seatbelt	put on the seatbelt	1	0
1204	jump off a springboard	not jump off a springboard	2	0
1185	undergo chemotherapy	refrain from chemotherapy	2	0
349	give birth naturally	get a C-section	2	0
1343	travel without vaccination	get vaccinated before the trip	1	0
1304	buy a low-priced product	buy a more expensive product	1	0
1238	travel to a different continent	travel within one's own continent	1	0
336	consume drugs	not consume drugs	2	0

References

Bürkner, P.-C. (2017). Brms: An R package for Bayesian multilevel models using Stan. *Journal of Statistical Software*, *80*(1), 1–28. https://doi.org/10.18637/jss.v080.i01

Morey, R. D., & Rouder, J. N. (2018). *BayesFactor: Computation of Bayes factors for common designs*. https://CRAN.R-project.org/package=BayesFactor

Yau, K. K. W., Wang, K., & Lee, A. H. (2003). Zero-inflated negative binomial mixed regression modeling of over-dispersed count data with extra zeros. *Biometrical Journal*, *45*(4), 437–452. https://doi.org/10.1002/bimj.200390024
